# Multiscale modeling of layer formation in epidermis

**DOI:** 10.1371/journal.pcbi.1006006

**Published:** 2018-02-26

**Authors:** Huijing Du, Yangyang Wang, Daniel Haensel, Briana Lee, Xing Dai, Qing Nie

**Affiliations:** 1 Department of Mathematics, University of Nebraska-Lincoln, Lincoln, NE, United States of America; 2 Department of Mathematics, University of California Irvine, Irvine, CA, United States of America; 3 Center for Complex Biological Systems, University of California Irvine, Irvine, CA, United States of America; 4 Department of Biological Chemistry, School of Medicine, University of California Irvine, Irvine, CA, United States of America; Oxford, UNITED KINGDOM

## Abstract

The mammalian skin epidermis is a stratified epithelium composed of multiple layers of epithelial cells that exist in appropriate sizes and proportions, and with distinct boundaries separating each other. How the epidermis develops from a single layer of committed precursor cells to form a complex multilayered structure of multiple cell types remains elusive. Here, we construct stochastic, three-dimensional, and multiscale models consisting of a lineage of multiple cell types to study the control of epidermal development. Symmetric and asymmetric cell divisions, stochastic cell fate transitions within the lineage, extracellular morphogens, cell-to-cell adhesion forces, and cell signaling are included in model. A GPU algorithm was developed and implemented to accelerate the simulations. These simulations show that a balance between cell proliferation and differentiation during lineage progression is crucial for the development and maintenance of the epidermal tissue. We also find that selective intercellular adhesion is critical to sharpening the boundary between layers and to the formation of a highly ordered structure. The long-range action of a morphogen provides additional feedback regulations, enhancing the robustness of overall layer formation. Our model is built upon previous experimental findings revealing the role of Ovol transcription factors in regulating epidermal development. Direct comparisons of experimental and simulation perturbations show remarkable consistency. Taken together, our results highlight the major determinants of a well-stratified epidermis: balanced proliferation and differentiation, and a combination of both short- (symmetric/asymmetric division and selective cell adhesion) and long-range (morphogen) regulations. These underlying principles have broad implications for other developmental or regenerative processes leading to the formation of multilayered tissue structures, as well as for pathological processes such as epidermal wound healing.

## Author summary

Epidermal morphogenesis, which occurs during the second half of embryogenesis, is the developmental process that generates a skin permeability barrier essential for terrestrial survival. Defects with this barrier are associated with common skin disorders such as atopic dermatitis. Study of mechanisms that control epidermal development and differentiation is therefore highly relevant to human health. Motivated by recent experimental observations on the role of Ovol transcription factors in regulating epidermal development, we developed a multiscale model to investigate the underlying mechanisms responsible for epidermal layer formation and homeostasis. We report that regulation of proliferation and differentiation by Ovol plays an important role in epidermal development. In addition, our computational analysis shows that asymmetric cell division, selective cell adhesion, and morphogen regulation work in a synergetic manner to produce the well-stratified epidermal layers. Taken together, our results demonstrate that robust epidermal morphogenesis involves a balance between proliferation and differentiation, and an interplay between short- and long-range spatial control mechanisms. This principle may also be applicable to other complex systems of tissue development or regeneration.

## Introduction

**Fig 1:**
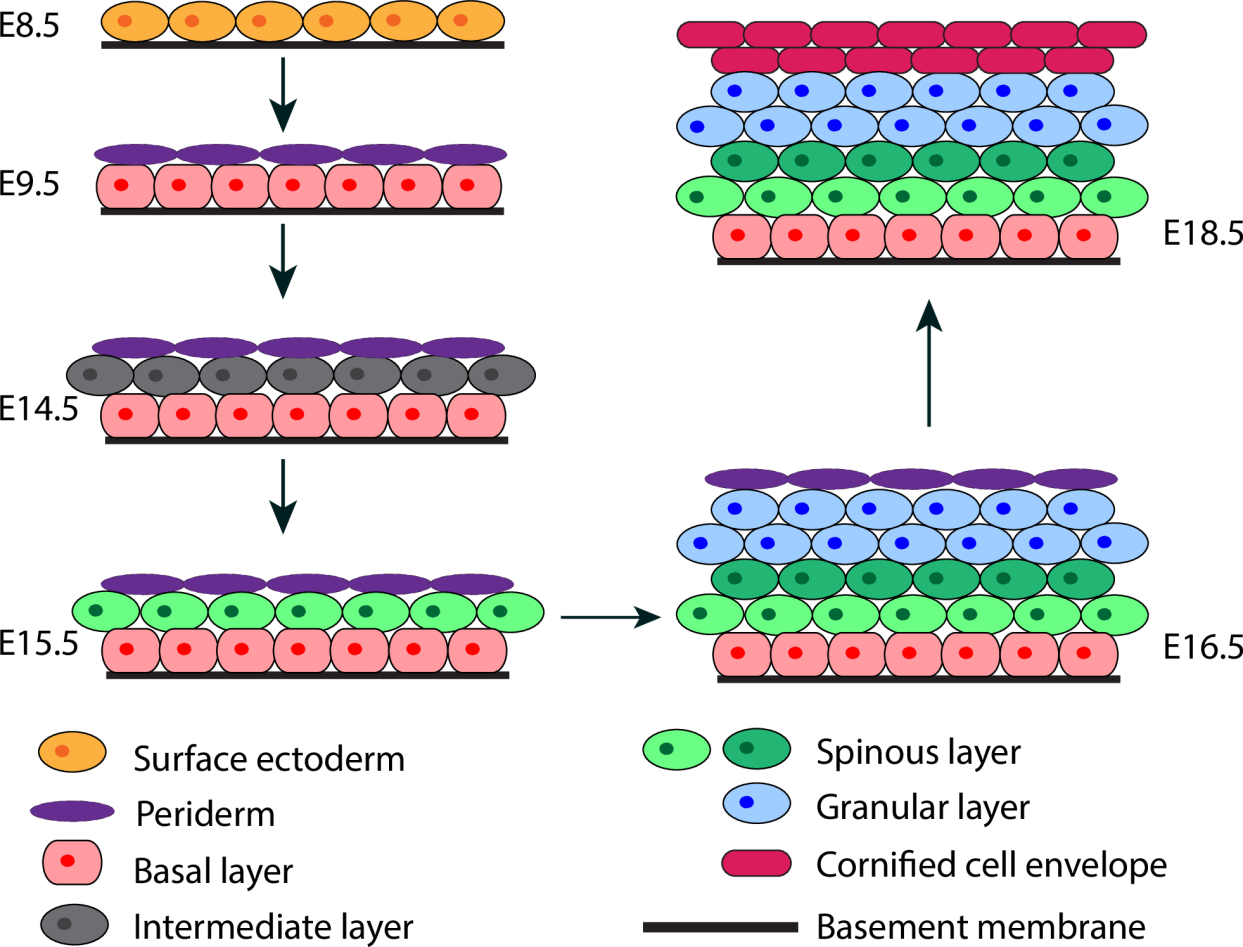
Schematic illustration of epidermal morphogenesis. Different layers are labeled in different colors.

Skin epidermis is a highly organized tissue that forms an essential barrier between an organism and its surrounding environment to protect the organism from dehydration, mechanical trauma, and microbial assaults. The mammalian epidermis is divided into four distinct compartments (from the innermost to the outermost): stratum basale (basal), stratum spinosum (spinous), stratum granulosum (granular), and stratum corneum (cornified) [1]. The formation of the epidermis is a complex yet robust process, relying on the coordinated regulation of a number of cellular events including but not limited to stem cell self-renewal, proliferation, cadherin-mediated cell-to-cell adhesion, integrin-mediated cell-to-basement membrane adhesion, differentiation, and migration [2–6]. Formation of the different layers of epidermis (i.e., the stratification process) occurs during embryonic development, ensuring the production of a functional barrier at birth. In mice, stratification occurs in several stages over a period of less than 10 days (Fig 1) [7]. First, cells of the single-layered surface ectoderm commit to an epidermal fate. The embryonic basal layer then gives rise to the periderm that covers the developing epidermis until the cornified cell layer is formed [7, 8]. The intermediate cell layer develops between the basal layer and the periderm. Development of the intermediate layer is associated with asymmetric divisions of embryonic basal keratinocytes, which occur perpendicularly to the basement membrane giving rise to one basal cell maintaining its attachment to the basement membrane and one suprabasal cell [3]. The intermediate cells are capable of transient proliferation, and the loss of this proliferative capacity is associated with the maturation of intermediate cells into spinous cells. Spinous cells subsequently undergo further differentiation into granular and cornified cells.

Transcriptional regulation is central to epidermal morphogenesis. Our previous studies have revealed important roles of Ovol transcription factors in epidermal proliferation and differentiation. Loss of Ovol1 in mice delays the cell cycle exit of epidermal progenitor cells during late embryogenesis [9], whereas loss of both Ovol1 and Ovol2 results in severe defects in epidermal development characterized by sluggish exit from a progenitor cell state, defective terminal differentiation, and failed barrier acquisition [10]. Conversely, transgenic overexpression of Ovol2 leads to precocious terminal differentiation at the expense of a progenitor cell compartment [10]. These findings provide a useful entry point to explore the strategic control of progenitor cell proliferation and differentiation during epidermal morphogenesis.

Mathematical modeling provides a powerful tool to reveal regulatory mechanisms that cannot be discovered by experiments alone. There are a number of modeling paradigms that can be utilized to model layer stratification. Previously, we developed continuum models to study the mouse olfactory epithelium, including a non-spatial model to explore how multi-lineage stages and feedback regulation govern tissue development and regeneration [11], as well as a one-dimensional (1D) spatial dynamic model of multi-stage cell lineages to study tissue stratification [12]. In such continuum models, cells are treated as a continuous density distribution, which provides a reliable approximation when large numbers of cells are present and individual properties of the cells are appropriately averaged. In systems such as the epidermis, where stratification entails proliferation and differentiation of a few layers of cells and their interactions with each other as well as the basement membrane, individual cell-based models are needed. A recent review has examined five popular individual-based approaches to model the self-organization of multicellular tissues [13]. For the epidermis, an agent-based modeling framework and a lattice-free cell-center method have been used to produce comprehensive 2D or 3D simulations for the spatiotemporal dynamics of epidermal homoeostasis [14–16]. An anisotropic subcellular element method was used to investigate the roles of complex cell morphology and biophysical anisotropic cell-cell interaction during basal-suprabasal layer formation [17]. These investigations focused on studying the roles of the basal layer stem cells in epidermal formation and homeostasis. Many questions surrounding the development from a single layer of stem cells to a stratified epithelia remain. For example, how do the different types of cells form layers with well-defined boundaries between them? When cells proliferate and differentiate, how do the different types of cells know where to go and when to start or stop to produce the correct size of different layers? How do Ovol, key transcription factors that regulate epidermal proliferation and differentiation, control the spatial organization of epidermal layers?

Here we present a 3D multiscale model of epidermis comprised of multiple cell types (basal, spinous, and granular) and incorporating regulation of cell proliferation and differentiation via a gene regulatory network. Major components in the 3D multiscale model include the discrete and stochastic Subcellular Element Model (SEM), which are used to describe individual cells, cell divisions, and physical cell-cell interactions. These models also incorporate dynamic systems such as gene regulatory network within each cells and a continuum model for extracellular morphogens. Specifically, symmetric and asymmetric cell divisions, selective cell-cell adhesion, diffusive signaling molecules, feedback regulations of extracellular morphogens, and cell lineage transitions are incorporated to allow natural emergence of multiscale interactions for layer formation. GPU algorithm is implemented to enable efficient parameter exploration.

Our study begins with analyzing known epidermal phenotypes of the *Ovol* mutant mice using both non-spatial and spatial models to interrogate the regulation of the epidermal cell lineage. We find critical regulations exerted by Ovol, namely inhibition of stem cell proliferation and stimulation of spinous cell differentiation, to be the most effective at recapitulating the experimentally observed epidermal phenotypes. Using this as a foundation, we expand our 3D multiscale model to examine the involvement of several spatial elements in epidermal morphogenesis. Our results show that short-range spatial mechanisms of symmetric/asymmetric division and selective cell adhesion in tandem with long-range regulation of the extracellular morphogen leads to robust epidermal stratification.

## Results

### Model description

We first consider a non-spatial cell lineage model [11, 18, 19] consisting of three different cell types with four stages: basal stem cells, proliferative intermediate spinous cells, mature non-proliferative spinous cells, and granular cells. Previous work has suggested that Ovol1 and Ovol2 repress the expression of one another [9, 10, 20]. Therefore we include an Ovo1-Ovol2 cross-repression loop in our model. In order to make a direct comparison with the Ovol perturbation data, we first explored various possible cellular effects of this loop, such as regulating the probability of stem cell self-renewal, proliferation rates, and maturation times of committed cells to obtain parameters that are consistent with the experimentally observed phenotypes of *Ovol*-deficient and -overexpressing mice (summarized in Table 1). To investigate interactions among three major biological scales: transcriptional regulations, individual cells, and the epidermis consisting of many cells and diffusive regulatory molecules secreted from the cells, we next develop a new 3D multiscale model that accounts for intracellular regulatory networks with noises, stochastic cell division, extracellular morphogens, individual cells, and cell population.

**Table 1:** Summary of observed phenotypes from wild type (WT), *Ovol1*-deficient (*Ovol1^-/-^*), *Ovol2*-deficient (*Ovol2* SSKO), *Ovol2*-overexpression (*Ovol2* BT), and *Ovol1/Ovol2*-deficient (*Ovol* DKO) mice.

	WT	*Ovol1*^-/-^	*Ovol2* SSKO	*Ovol2* BT	*Ovol* DKO
K14^+^ basal layer	—	—	—	—	↑
K1^+^ spinous layer	—	↑	—	↓	↑
Lor^+^ granular layer	—	—	—	↓	↓
*Ovol1* expression	*a*	0	*d*	*g*	0
*Ovol2* expression	*b*	*c*	0	*h*	0
Relations		*c>b*	*d>a*	*g<a*, *h>b*	

The symbols “↑”, “↓”, and “—” indicate an increase, decrease, and no change in layer size in comparison to the wild type, respectively. Relations between certain Ovol levels are derived from a mutual inhibition of *Ovol1* and *Ovol2* expression. K14, keratin 14; K1, keratin 1; Lor, loricrin.

#### Four-stage non-spatial lineage models

**Fig 2:**
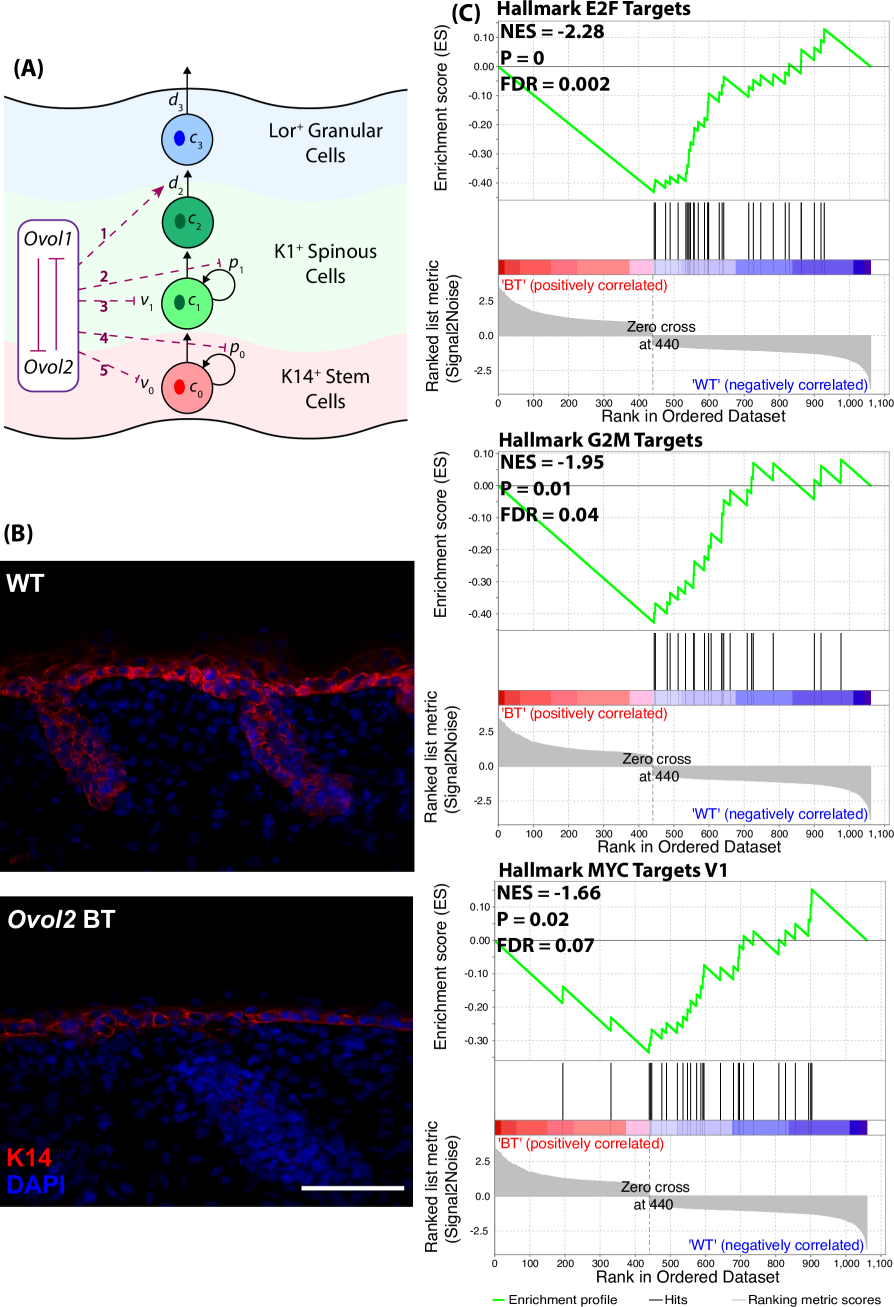
(**A**) A schematic diagram shows the roles of Ovol proteins in epidermal development and differentiation. Red dashed lines represent the potential regulation exerted by Ovol on components of the cell lineage model. (**B**) Immunofluorescence images showing a mild decrease in the size of the K14-positive compartment in *Ovol2*-overexpressing epidermis at E18.5. Scale bar represents 50 *μ*m. (**C**) GSEA of microarray data from E17.5 embryos (2 biological replicates per genotype) with Normalized Enrichment Score (NES), p-value (P), and False Discovery Rate (FDR).

The epidermal cell population is simplified into a cell lineage of three different cell types with four stages (Fig 2A): basal stem cells (represented as *C*_0_), proliferative spinous cells (*C*_1_), mature spinous cells (*C*_2_), and granular cells (which represent a terminally differentiated cell type in our model, represented as *C*_3_), respectively. Here, *p*_0_ is the probability for basal cells to self-renew upon division while 1 − *p*_0_ is the probability of symmetric differentiation to become proliferative spinous cells. *p*_1_ is the probability for proliferative spinous cells to stay proliferative upon division while 1 − *p*_1_ is the probability of exiting the cell cycle. *d*_2_ is the rate at which mature spinous cells differentiate into granular cells. *d*_3_ is the rate at which granular cells are removed from the system, which may occur through natural cell death or by entering into the stratum corneum. *v*_0_ and *v*_1_ are proliferation rates of basal and proliferative spinous cells, respectively, and take the form ln2/(cell cycle length) throughout the context of this model.

The equations governing the size of the cell populations in the epidermis are

$$\begin{array}{l} \frac{dc_{0}}{dt}=\left(2p_{0}-1\right)v_{0}c_{0},\\ \frac{dc_{1}}{dt}=2\left(1-p_{0}\right)v_{0}c_{0}+\left(2p_{1}-1\right)v_{1}c_{1},\\ \frac{dc_{2}}{dt}=2\left(1-p_{1}\right)v_{1}c_{1}-d_{2}c_{2},\\ \frac{dc_{3}}{dt}=d_{2}c_{2}-d_{3}c_{3}. \end{array}$$ (1)

Stability analysis of Eq 1 based upon experimental perturbations of *Ovol* (*Ovol*-deficient and -overexpressing mice; see S1 Text) has suggested that Ovol’s down-regulation of either *p*_0_ or *v*_0_ has the potential to explain the observed changes (or lack of changes) in basal layer size and that Ovol’s up-regulation of *d*_2_ may explain the observed changes in spinous and granular layer sizes in *Ovol* mutant skin. Accordingly, we present the following model that Ovol1 and Ovol2 inhibit *v*_0_ and *p*_0_ and up-regulate *d*_2_ through the following forms,

$$\begin{array}{l} v_{0}=v_{\min}+\frac{v_{T}}{1+\omega \alpha +\chi \beta },\\ p_{0}=p_{\min}+\frac{p_{T}}{1+\lambda \alpha +\mu \beta },\\ d_{2}=d_{DKO}+\varsigma \alpha +\xi \beta , \end{array}$$ (2)

where *α* and *β* denote the intracellular levels of Ovol1 and Ovol2, respectively. We assume that Ovol1 and Ovol2 inhibit the expression of one another as described above.

#### Spatial multiscale model consisting of discrete cells in 3D and continuum calcium distribution

The 3D multiscale framework includes three major components: (1) the SEM for individual cell dynamics, cell morphology, cell growth, cell proliferation, and cell-to-cell contact and communications; (2) continuum reaction-diffusion-advection (RDA) partial differential equations (PDEs) for extracellular morphogen production, diffusion and transportation; (3) cell lineage transition for differentiation regulated by a number of feedback loops, e.g. via the probability of basal stem cell self-renewal, proliferation rates and maturation times of committed cells.

The discrete cell model includes (i) intracellular forces that determine cell shape and polarity, (ii) cell-type-dependent intercellular adhesion forces that control cell-cell interactions, and (iii) external forces that provide cell-basement membrane adhesion. Each cell is constructed from a number of discrete subcellular elements. The model is built upon our previous 2D model [17] which focuses only on the basal layer, and our previous 3D model on crypts [21]. Elements of different cell types are assigned different pairwise intercellular forces that accounts for selective cell adhesion. Two types of intercellular adhesion strength are defined: *F*_*a*_ for the same type cells and *F*_*b*_ for the different type cells. It’s assumed that adhesion strength is different among cell types, and the adhesion force within the same cell type is greater than that between different cell types (*F*_*a*_ > *F*_*b*_ for each cell). Additionally, each cell is comprised of at least two element types with different properties, and different element types interact differently with the environment through element type-dependent intercellular and external forces (see Materials and Methods for details).

Cell growth is modeled as an element that is added to each cell at regularly scheduled intervals, resulting in a volume increase similar to our previous approach [21]. Upon division, the levels of Ovol1 and Ovol2 regulate the proliferation and differentiation rates of basal cells and spinous cells. Both regulators reduce the rates at which their target cells divide, and increase the probability that the products of those divisions differentiate into cells of the next lineage stage (Eq 2). Cells divide symmetrically into two daughter cells of the same type by a division plane roughly perpendicular to the basement membrane. Cells divide asymmetrically into two daughter cells of two different cell types by a division plane roughly parallel to the basement membrane. When a granular cell reaches the cell cycle length, this cell will be removed from the system: Elements within this cell are removed gradually in a time interval, resulting in volume shrinking, and finally, removal of the entire cell altogether.

Calcium was chosen as the morphogen of interest in our model given its widely recognized role in triggering epidermal differentiation [9, 10, 22–24]. In the *Ovol1/2* double knockout model, calcium induces less remarkable differentiation of the mutant epidermal keratinocytes [10]. Calcium distribution displays a gradient that rises from low levels in the basal layer to a peak at the upper granular layer, and drops sharply to low levels in the cornified layer [25]. Thus, in our model each element of mature spinous and granular cells secret calcium to the environment. Diffusion-reaction systems are used to capture the chemical kinetics among cell layers:

$$\frac{\partial s}{\partial t}=D_{s}\Delta s+f\left(s,c_{2},c_{3}\right)-d_{s}s,$$ (3)

where *s* denotes the calcium concentration on a given domain, *D*_*s*_ is the diffusion coefficient, and *d*_*s*_ is the decay rate. This chemical field is simulated on the regular grid, and a reverse mapping is used to determine the value of this signal field that each cell is exposed to. The second term on the right hand side, *f*(*s*, *c*_2_, *c*_3_), represents the total secretion of calcium, where *c*_2_ and *c*_3_ represents the number density of mature spinous and granular cells, respectively, mapping from subcellular element model to PDE grid. We assume that each cell secretes a constant amount of signal into the grid, and the secretion rate is *δ*_*s*1_ and *δ*_*s*2_ for mature spinous and granular cells, respictively. The production function for calcium is *f*(*s*, *c*_2_, *c*_3_) = *δ*_*s*1_*c*_2_ + *δ*_*s*2_*c*_3_.

### Coordination of cell proliferation and differentiation controls the proportions of different types of cells

In previously published experiments, genetic deletion of *Ovol1/Ovol2* or overexpression of *Ovol2* resulted in an epidermal tissue with altered size and abnormal proportions of different epidermal cell types [9, 10, 23] (Table 1). To investigate how embryonic epidermis achieves appropriate stratification, a four-stage cell lineage model (Eq 1) similar to [18, 19] was used to explore the transcriptional mechanisms governing growth and differentiation incorporating the Ovol transcription factors, and the model is constrained by the experimental data.

Using the cell lineage model, we first evaluated the sensitivity of the different epidermal layers with respect to the cellular parameters in Eq 1 (details in S1 Text). Based on the skin phenotypes of gain- and loss-of function *Ovol* mutants in Table 1, we hypothesized that Ovol1 and Ovol2 may down-regulate *p*_0_ (i.e., promote basal cell to spinous cell transition), *p*_1_ (i.e., promote growth arrest of spinous cells); *v*_0_ (i.e., inhibit the proliferation rate of basal cells), *v*_1_ (i.e., inhibit the proliferation rate of spinous cells), and/or up-regulate *d*_2_ (i.e., promote terminal differentiation of progenitor cells into granular cells) (red dashed lines in Fig 2A, and see S1 Text for more details).

By exploring the various possible effects of the Ovol regulation loop on the dynamics of cell lineages (red dashed lines in Fig 2A), we found that the simplest form of lineage regulations which can recapitulate the *in vivo* epidermal phenotypes of all four *Ovol* mutants, is Model 1 (dashed lines 1+4) where Ovol proteins inhibit *p*_0_ and stimulate *d*_2_, or Model 2 (dashed lines 1+5) where Ovol proteins inhibit *v*_0_ and stimulate *d*_2_ (see Table 2 and S1 Text). More complicated models with additional regulators (dashed lines 2 or 3 in Fig 2A) to Model 1 or Model 2 are also able to recapitulate the epidermal phenotypes (see S1 Text). Overall, the modeling studies suggests that Ovol1 and Ovol2 exert pleiotropic effects with two key components: decreased epidermal stem/progenitor cell self-renewal/proliferation and increased terminal differentiation.

**Table 2:** Results from simulations with Ovol regulation can recapitulate the epidermal phenotypes of all mutants. The model details and parameter values are shown in S1 Text.

***Model 1***	**WT**		***Ovol1***^**-/-**^		***Ovol2* SSKO**		***Ovol2* BT**		***Ovol* DKO**	
K14^+^ basal layer	1.60	—	1.87	—	1.72	—	1.13	—	4.06	↑
K1^+^ spinous layer	8.18	—	17.50	↑	8.37	—	3.08	↓	169.14	↑
Lor^+^ granular layer	4.11	—	4.56	—	4.38	—	3.07	↓	1.55	↓
*Ovol1* (α)	*a* = 1		0		*d* = 1.6		*g* = 0.5		0	
*Ovol2* (β)	*b* = 1		*c* = 1.25		0		*h* = 10		0	
***Model 2***	**WT**		***Ovol1***^**-/-**^		***Ovol2* SSKO**		***Ovol2* BT**		***Ovol* DKO**	
K14^+^ basal layer	2.81	—	2.90	—	2.85	—	2.59	—	3.35	↑
K1^+^ spinous layer	13.37	—	25.78	↑	13.17	—	4.27	↓	174.80	↑
Lor^+^ granular layer	6.57	—	6.59	—	6.75	—	5.80	↓	1.61	↓
*Ovol1* (α)	*a* = 1		0		*d* = 1.6		*g* = 0.5		0	
*Ovol2* (*β*)	*b* = 1		*c* = 1.25		0		*h* = 50		0	

Consistent with Models 1 and 2, our previous experimental work has suggested a role for Ovol1 and Ovol2 in suppressing an epidermal progenitor cell fate while facilitating their terminal differentiation [10]. In particular, the number of mitotic cells in the basal layer increases from wild-type and *Ovol1*^*-/-*^ to *Ovol* DKO epidermis [10]. To seek further supporting evidence for a specific effect of Ovol2 on epidermal basal cells, we stained skin from *Ovol2*-overexpressing mice [10] with basal cell marker K14. A significant reduction in the size of the K14-positive compartment was indeed observed upon *Ovol2* overexpression (Fig 2B), supporting the notion that Ovol2 may suppress *p*_0_ or *v*_0_. Moreover, gene set enrichment analysis (GSEA) of microarray data obtained from skin of *Ovol2*-overexpressing and control embryos [10] revealed that gene signatures associated with proliferation and cell cycle are de-enriched upon *Ovol2* overexpression, supporting the possibility that Ovol2 suppresses *v*_0_ (Fig 2C). Together, these experimental findings provide validation for our model, and lay the foundation for us to next explore the model to assess the contribution of additional cellular and molecular processes to robust epidermal stratification.

### Stem cell asymmetric division and polarized cell adhesion together lead to robust basal-suprabasal boundary formation

**Fig 3:**
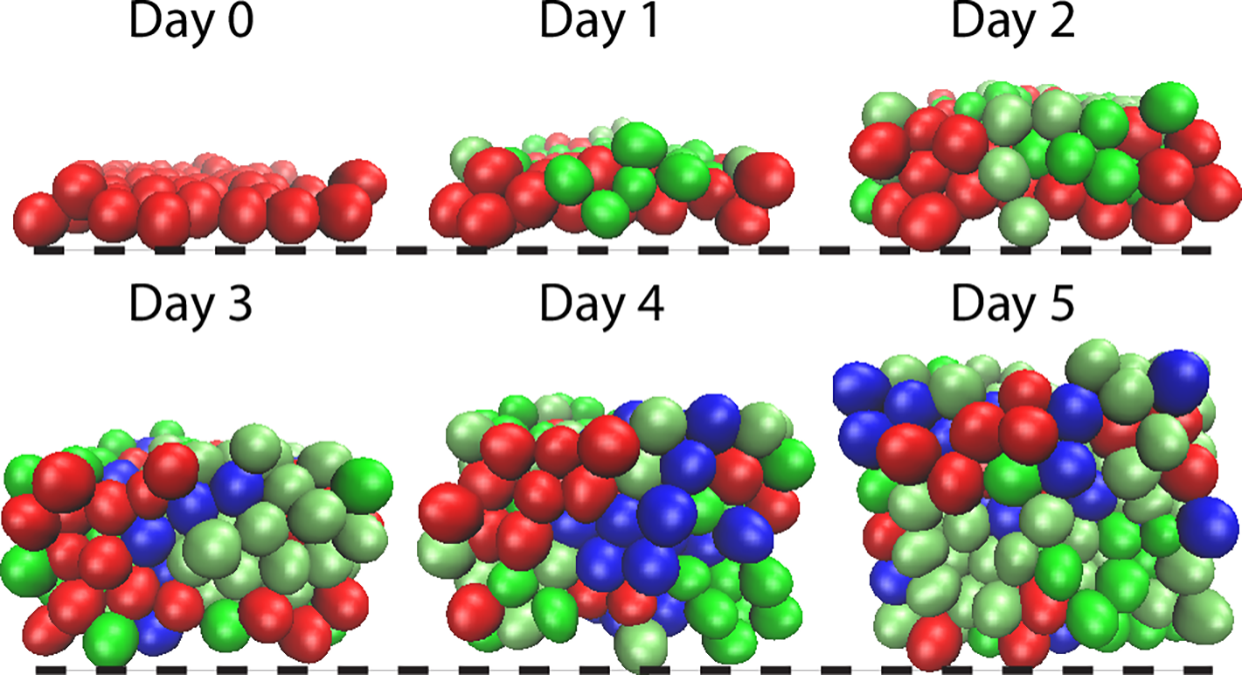
Time course images of layer formation from Base Model (with cell lineage information but without spatial regulation) exhibit highly heterogeneous distribution of cells. All the parameters used in the simulations are in Table A in S2 Text.

Next we incorporated the four-stage cell lineage model into a 3D multiscale model that only includes cell proliferation and differentiation (and thus denoted Base Model). Table 3 shows that this Base Model, which involves Ovol’s inhibition of *v*_0_ or *p*_0_ and stimulation of *d*_2_, is also able to recapitulate the epidermal phenotypes, particularly the alterations in the numbers of various cell types, that result from genetic manipulations of *Ovol*. As expected, however, without additional spatial regulation, epidermal formation in the Base Model exhibits highly heterogeneous distribution of cells in the layers resulting in strong stochastic variation (Fig 3).

**Table 3:** 3D simulation results with Ovol down-regulation of *v*_0_ and *p*_0_ and up-regulation of *d*_2_ can recapitulate the epidermal phenotypes of all mutants, in cell number wise. Mean and standard deviation of the cell number in 3D model is over an ensemble of 20 simulations. The model details and parameter values are shown in S2 Text.

*Model*		WT	*Ovol1*^-/-^		*Ovol2* SSKO		*Ovol2* BT		*Ovol* DKO	
3D hybrid	*C*_0_	285±34	305±32	—	280±39	—	277±36	—	330±24	↑*
	*C*_1_+*C*_2_	1199±178	4973±539	↑**	1086±136	—	706±96	↓**	21256±1262	↑**
	*C*_3_	748±92	680±51	—	816±83	—	620±66	↓*	122±18	↓**

* represents p-value<0.005 for t-test

** represents p-value<0.00001.

**Fig 4:**
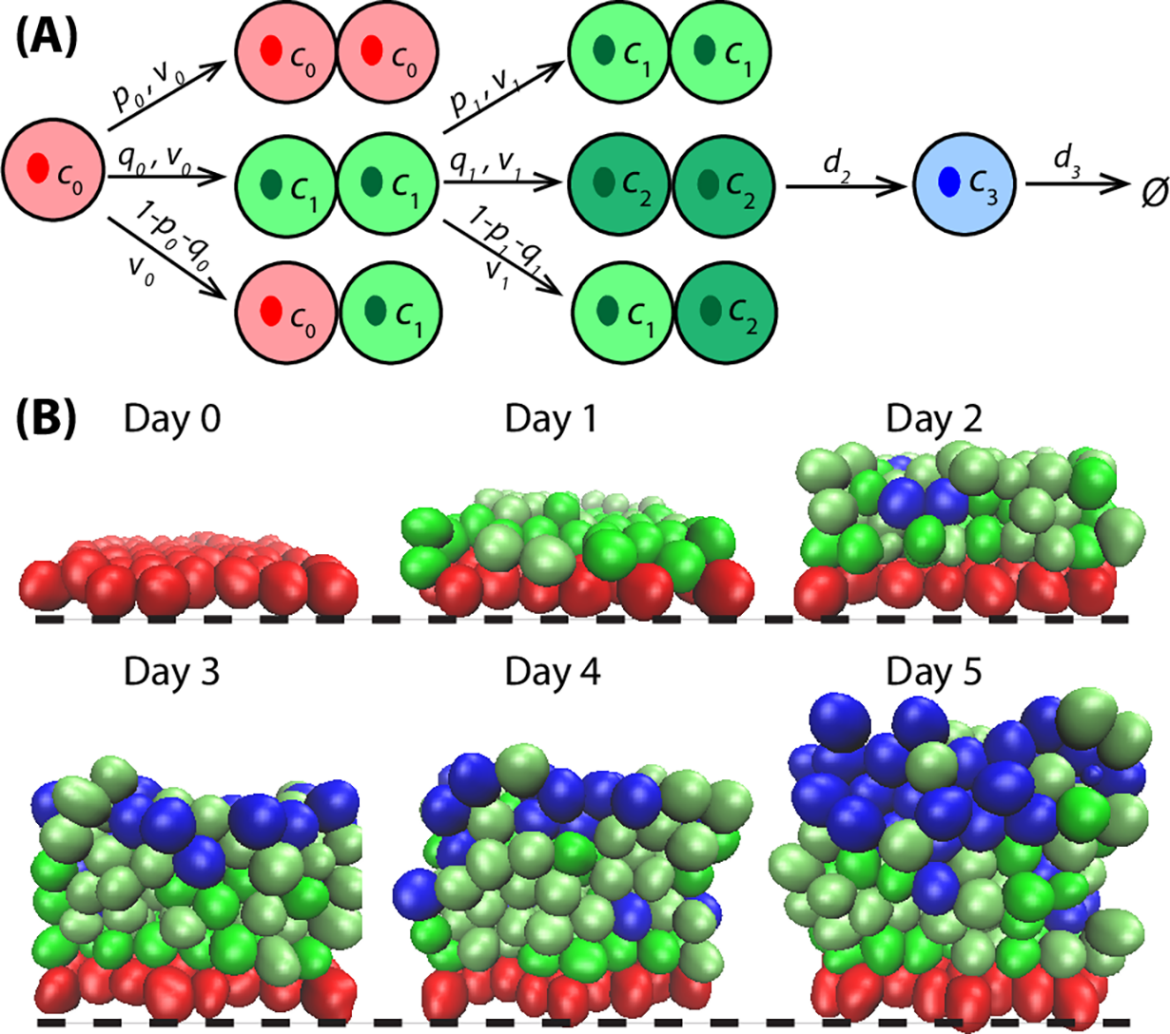
Basal cell asymmetric division and polarized cell adhesion together control robust basal-suprabasal boundary formation. (**A**) A schematic lineage diagram shows the asymmetric cell division. (**B**) Time course images of layer formation from Asymmetric Division Model. Black dashed lines represent the basement membrane. All the parameters used in the simulations are in Table A in S2 Text.

Previous work has demonstrated that polar distribution of cell-cell and cell-substrate adhesions coupled with a developmental switch to asymmetric division leads to robust, predictable stratification between the basal and suprabasal cells [17]. Following this idea, the spatial regulation of asymmetric division and polarized cell adhesion is implemented into the 3D Base Model (Fig 4A), and this is referred to as Asymmetric Division Model. Basal cells undergo asymmetric division to produce a basal stem cell and a spinous cell, with the latter naturally assuming a suprabasal position [3]. If there is only symmetric division, the perpendicular division plane will produce two daughter cells side by side in basal layer, which will grow and compete for the limited space, yielding large contact pressure and possibly dramatic variation of the neighboring layer formation. A comparison of time course images (Fig 4B) shows that asymmetric division increases the natural stratification between basal stem and spinous cells, yielding a clear boundary between the two cell types.

**Fig 5:**
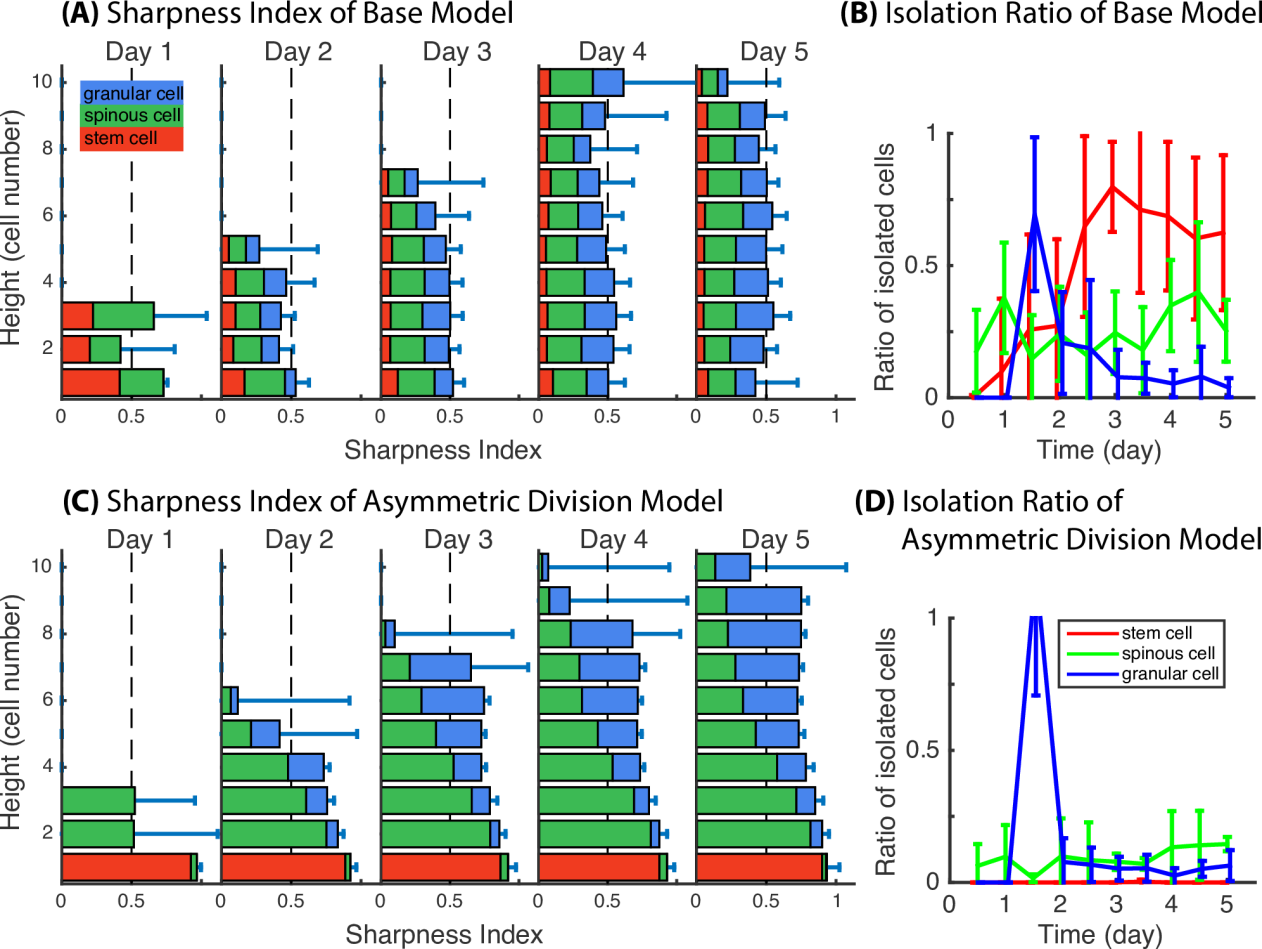
Comparison in Sharpness Index and Isolation Ratio demonstrate the quantitative proof of improved layer formation with asymmetric cell division and polarized cell adhesion. (**A,C**) Sharpness Index for Base Model and Asymmetric Division Model. Bar length represents the value of Sharpness Index at various height in the unit of cell numbers. Red, green and blue stack bars represent the portion of basal, spinous and granular cells at each slice. (**B,D**) Isolation Ratio for Base Model and Asymmetric Division Model. Mean and standard deviation in 3D model are over an ensemble of 20 simulations. All the parameters used in the simulations are in Table A in S2 Text.

To quantitatively assess epidermal stratification, we introduced two measurements (see Materials and Methods): Sharpness Index, which resembles 1 minus the standard deviation within each slice along the z-axis, and Isolation Ratio, which quantifies the proportion of cells that are far away from their own target layer for each cell type. Time course evolution of Sharpness Index and Isolation Ratio for the Base Model and the Asymmetric Division Model is presented in Fig 5. In the Base Model, the Sharpness Index over time is less than or equal to 0.5, and the stacked bars for cell type proportion are evenly distributed along z-axis, indicating a stochastically well-mixed layer due to lack of spatial regulation in layer formation. In the Asymmetric Division Model, the Sharpness Index is overall improved to be greater than 0.7 for most layers, and the red stack bars representing the basal stem cell proportion are restricted to the basal layers. This clearly shows that with incorporation of asymmetric division, the stratification level significantly increases for the basal and proliferative spinous layers of the epidermis. With basal cells being restricted to the basal layer and not intermingling with spinous and granular cells of the tissue, the Sharpness Index of spinous and granular layers consequently increases. The observation of a decreased Isolation Ratio is consistent with the notion that basal stem cells stay within the basal layer, and spinous and granular cells migrate closer to their own target locations. A drawback to the Asymmetric Division Model is the introduction of stochastic perturbations during layer formation with spinous and granular cells passively transported upwards from the lower layers by proliferation pressure. This is also captured with a decreased Sharpness Index due to the intermingling of spinous and granular cells, as well as a relatively higher Isolation Ratio of the two cell types. To further analyze the spatial pattern of the same type of cells at multiple distances, we calculated another measure of spatial structure: Ripley’s *K* function [26] in each slice along the z direction. We compared a typical simulation from the Base Model (Fig C in S2 Text) and the Asymmetric Division Model (Fig D in S2 Text), and found that the Ripley’s *K* function calculations show consistent results for the spatial distribution of the different cell types.

### Selective cell adhesion is critical for boundary formation between the different cell types

**Fig 6:**
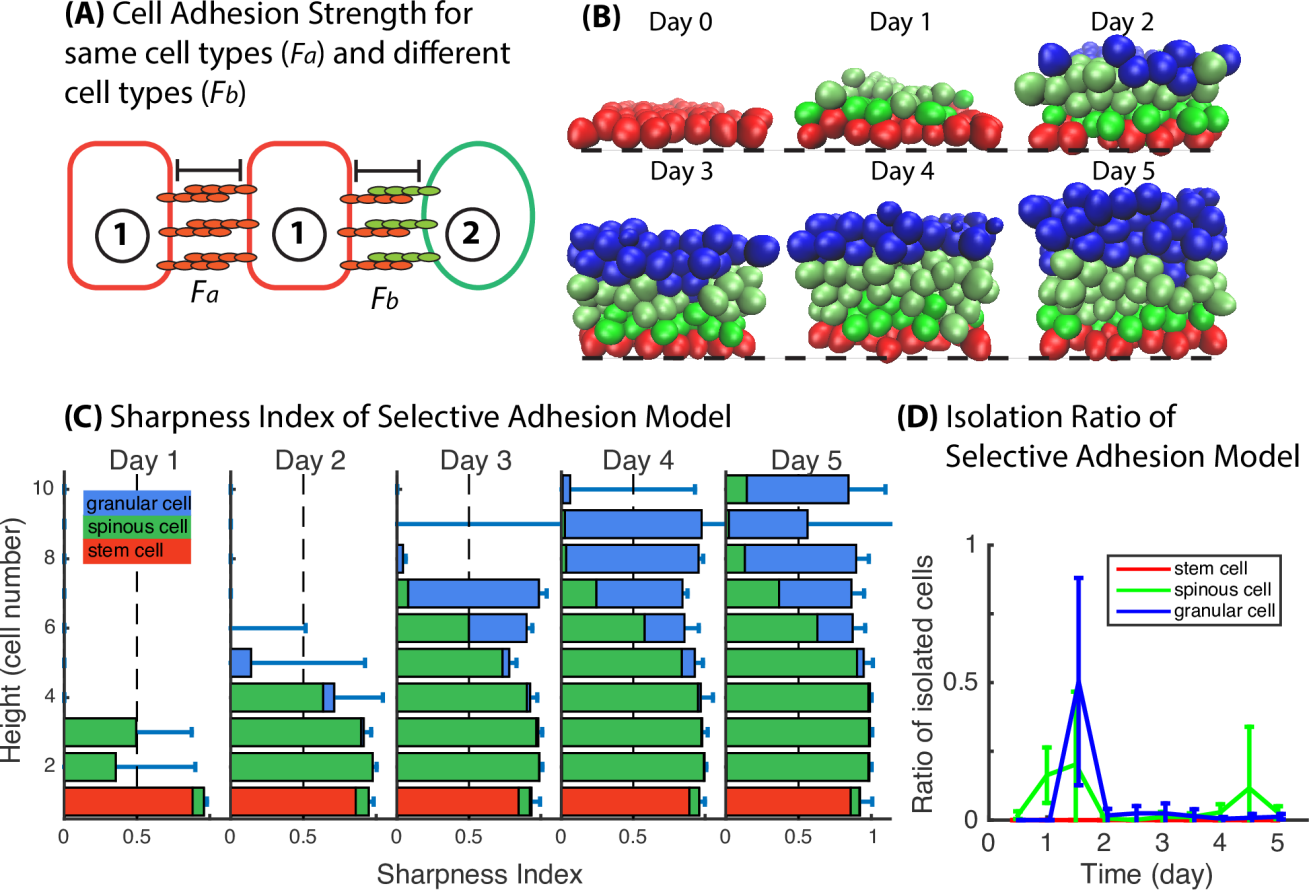
Selective cell adhesion significantly sharpens the boundaries between spinous and granular layers. (**A**) A schematic diagram shows the selective cell adhesion mechanism. (**B**) Time course images of layer formation of Selective Adhesion Model. Black dashed lines represent the basement membrane. (**C**) Sharpness Index. Bar length represents the value of sharpness index at height in the unit of cell numbers. Red, green and blue stack bars represent the portion of basal, spinous and granular cells at height in the unit of cell numbers. (**D**) Isolation Ratio. Mean and standard deviation in 3D model is over an ensemble of 20 simulations. All the parameters used in the simulations are in Table A in S2 Text.

In our model, cell interactions are represented through the potential function between subcellular elements. Subcellular elements both within and between cells will be mutually repulsive if their separation is below the equilibrium size of an element. For separations larger than this size, the elements will be mutually attractive, but with the strength of attraction falling off rapidly with separation. Evolution of the system is then prescribed by a large coupled system of Langevin equations for all elements. When the same adhesion strength is assigned to all cell types, the intracellular force will always be isotropic. In order for the reorganization among spinous and granular cells to occur, differential adhesive strength between different cell types is needed. Two types of intercellular adhesion strength are defined: *F*_*a*_ for the same type cells and *F*_*b*_ for the different type cells (Fig 6A). We assume that adhesion strength is different among cell types, and the adhesion force within the same cell type is greater than that between different cell types, i.e., *F*_*a*_ > *F*_*b*_. Initially, we set *F*_*a*_ = 4*F*_*b*_. To restrict intercellular adhesion within short range, the adhesion strength falls to zero once the cell distance is beyond a two-cell diameter. This model is indicated as Selective Adhesion Model.

The time course snapshots show that the proliferative spinous cells, the mature spinous cells and the terminally differentiated granular cells do not intermingle as long as selective cell adhesion is maintained (Fig 6B). The quantitative analysis (Fig 6C and 6D) showing improvements in Sharpness Index for the spinous and granular layers of the tissue together with reduced Isolation Ratio confirms that selective cell adhesion between epithelial cells, introduced by the differential activation of cadherin receptors and ligands, is necessary to regulate layer formation. The calculation of Ripley’s *K* function of a typical simulation with the stratified tissue (Fig E in S2 Text) shows that the different types of cells are separated into different layers and that the cells distribute regularly within their own layer.

To examine the performance of selective cell adhesion in detail, snapshots from several typical 3D simulations are presented. Most simulations end up with a clearly stratified layer structure (Fig 6B). Some simulations fail to form a proper stratified epithelium, specifically in the differentiated cell types (Fig B in S2 Text). In some scenarios, the thickness of differentiated cell layers shows large variation (Fig B in S2 Text, scenarios 1,2). With stochastic effects, this variation decreases in certain cases with time evolution leading to even and flat layers, but in some cases, variation increases leading to unconnected pieces of spinous or granular cells (scenarios 2,3,4 in Fig B in S2 Text). A corresponding Ripley’s *K* function calculation (Fig F in S2 Text) shows the similar results that there exist a cluster of spinous cells above the granular layer. Such failures reflect the limitation of selective cell adhesion in two ways. First, selective adhesion tends to make the same type of cells form clusters with spherical shape, and these spherical cell clusters break the flat layer pattern (scenarios 1,2,4 in Fig B in S2 Text). Second, selective cell adhesion is a short-range mechanism, in simulation we assume that attraction between cell elements drops to zero when they are farther than two-cell diameter length, as a result the isolated cells, which are far from their target layer and endure very small even no attraction force that would lead them to join their target layer, will stay where they are for a long time until they differentiate (scenarios 3,4 in Fig B in S2 Text). For example in scenario 3 (Fig B in S2 Text), granular cell, trapped within the spinous layer, will either be removed from the system at the end of its cell cycle, or slowly migrate upwards into granular layer by proliferation pressure or intercellular interaction, and scenario 3 will get resolved within half cell cycle time (12 hours). In scenario 4 (Fig B in S2 Text), when spinous cells are trapped above the granular layer, they are far from the spinous layer and experience no attraction from other spinous cells, therefore they will stay at the top for a very long time (about two cell cycle time, ≈48 hours). Although most simulations end up with normal stratified epidermis as in Fig 6, once the scenarios in Fig B in S2 Text occur, selective cell adhesion is no longer able to or takes a very long time to resolve this issue.

**Fig 7:**
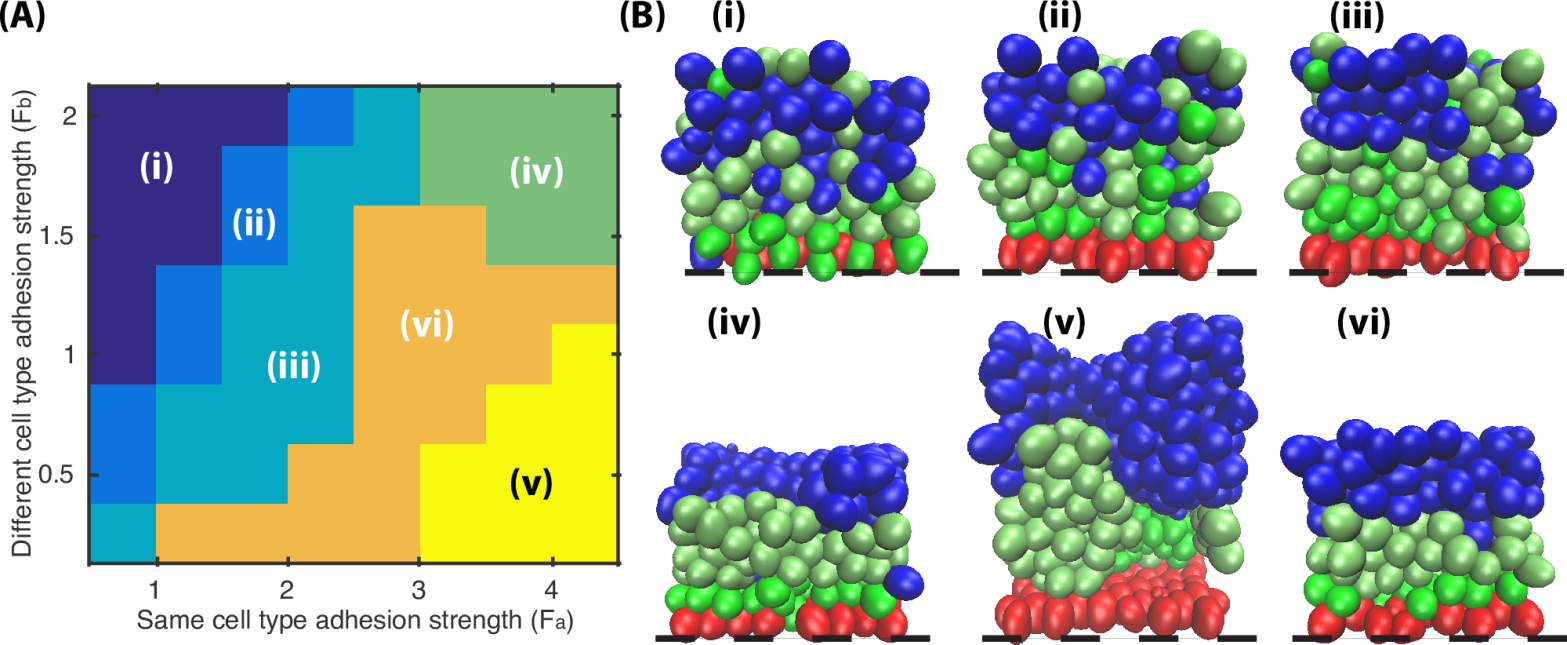
Effectiveness of selective cell adhesion based on varied adhesion strength *F*_*a*_ and *F*_*b*_. The optimal results shows the selective cell adhesion works most efficiently when ratio *F*_*a*_/*F*_*b*_ is between 2 and 6 (Pattern vi). All the parameters used in the simulations are in Table A in S2 Text.

To investigate the effect of adhesion strength, *F*_*a*_ and *F*_*b*_ are varied between 0.5 and 5 while other parameters are kept the same. When *F*_*a*_ < *F*_*b*_, the effect of selective cell adhesion is reversed, and the distribution of spinous and granular cells shows a salt-pepper pattern (Fig 7, Pattern i). When *F*_*a*_ = *F*_*b*_, cell adhesion is isotropic and the resulting layer formation resembles the results obtained with the Asymmetric Division Model (Fig 7, Pattern ii). When *F*_*a*_ > *F*_*b*_ and they are set at relatively small values, the effect of selective adhesion is not strong enough to reorganize the mixed layers (Fig 7, Pattern iii). When *F*_*a*_ > *F*_*b*_ and both are set to be relatively large values, the strong intercellular adhesion largely reduces the tissue volume, the layers are compact, and the freedom of isolated cells is highly constrained (Fig 7, Pattern iv). When *F*_*a*_ > *F*_*b*_ and *F*_*a*_ is set to be relatively large, the same type of cells move extensively and the clusters are prone to detach from each other, generating a large inner cavity within the aggregate (Fig 7, Pattern v). Only when *F*_*a*_ > *F*_*b*_ and the ratio of *F*_*a*_/*F*_*b*_ is bounded within a zone (Fig 7, Pattern vi), the simulations yield well-stratified epidermis. Combined, simulations of varied adhesion strength suggests that the selective cell adhesion works most efficiently when ratio *F*_*a*_/*F*_*b*_ is between 2 and 6 (Fig 7, Pattern vi), which allows cells not only enough energy to progressively generate and maintain stratified layers but also flexibility to get rid of isolation scenarios.

**Fig 8:**
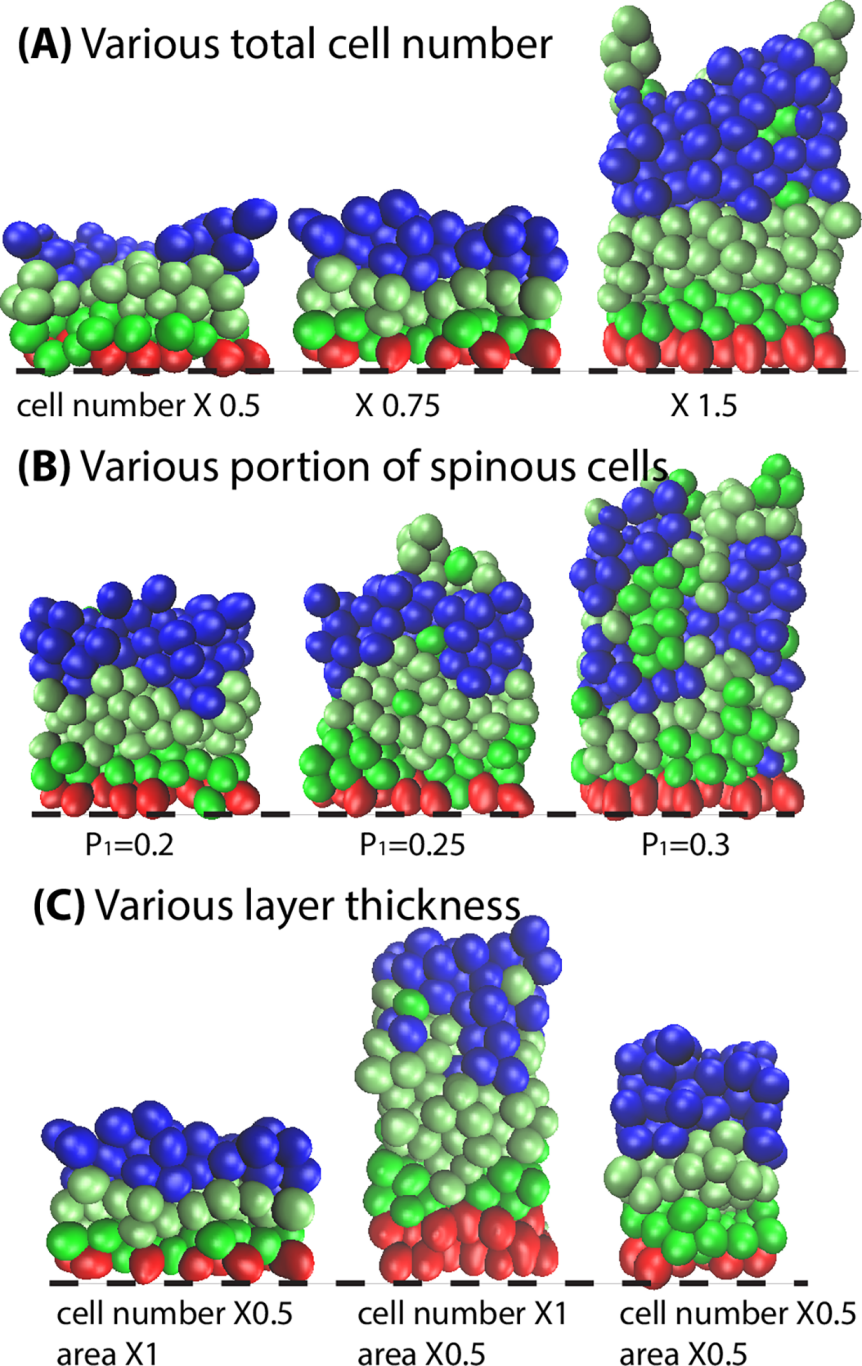
Effectiveness of selective cell adhesion based on various cell number and layer thickness. The mechanism performs most efficiently when the cell layer thickness is around or below 4 cell diameter thick. All the parameters used in the simulations are in Table A in S2 Text.

To better investigate the short-range feature of selective cell adhesion, we began simulations by varying the total cell number within the epidermis. The simulation snapshots thus obtained are summarized in Fig 8A. When the total cell number is slightly decreased (X0.75), although consequently the cell number in each cell type decreases and the thickness of the corresponding layers shrinks, layer formation is still maintained. When the total cell number is further decreased to a greater extent (X0.5), the reduction in cell number results in the formation of cavities among the different layers. On the other hand, when the total cell number is increased (X1.5), the layer thickness for each cell type increases accordingly. Once the thickness of granular layer is greater than 4 cell diameter, the isolated spinous cells above the granular layer are not able to, or take a very long time to, return to the spinous layer. Instead of artificially altering the total cell numbers, we adjusted the proliferation rate *p*_1_ (Fig 8B). When *p*_1_ is increased from 0.2 to 0.3, the total number of spinous cells increases accordingly leading to a thickened spinous layer, which greatly decreases layer formation. To investigate the effect of various layer thickness on selective cell adhesion, we changed the total cell number (Fig 8C). With decreased cell number, the stratified layer formation was still maintained. In general, due to the short-range feature of selective cell adhesion, this mechanism works under the restriction of appropriate cell number and layer thickness, generating an appropriately stratified epidermis when the cell layer thickness is around or below a certain range (4 cell diameter thick).

### Long-range signaling enhances spatial organization and the size of spinous and granular layers

**Fig 9:**
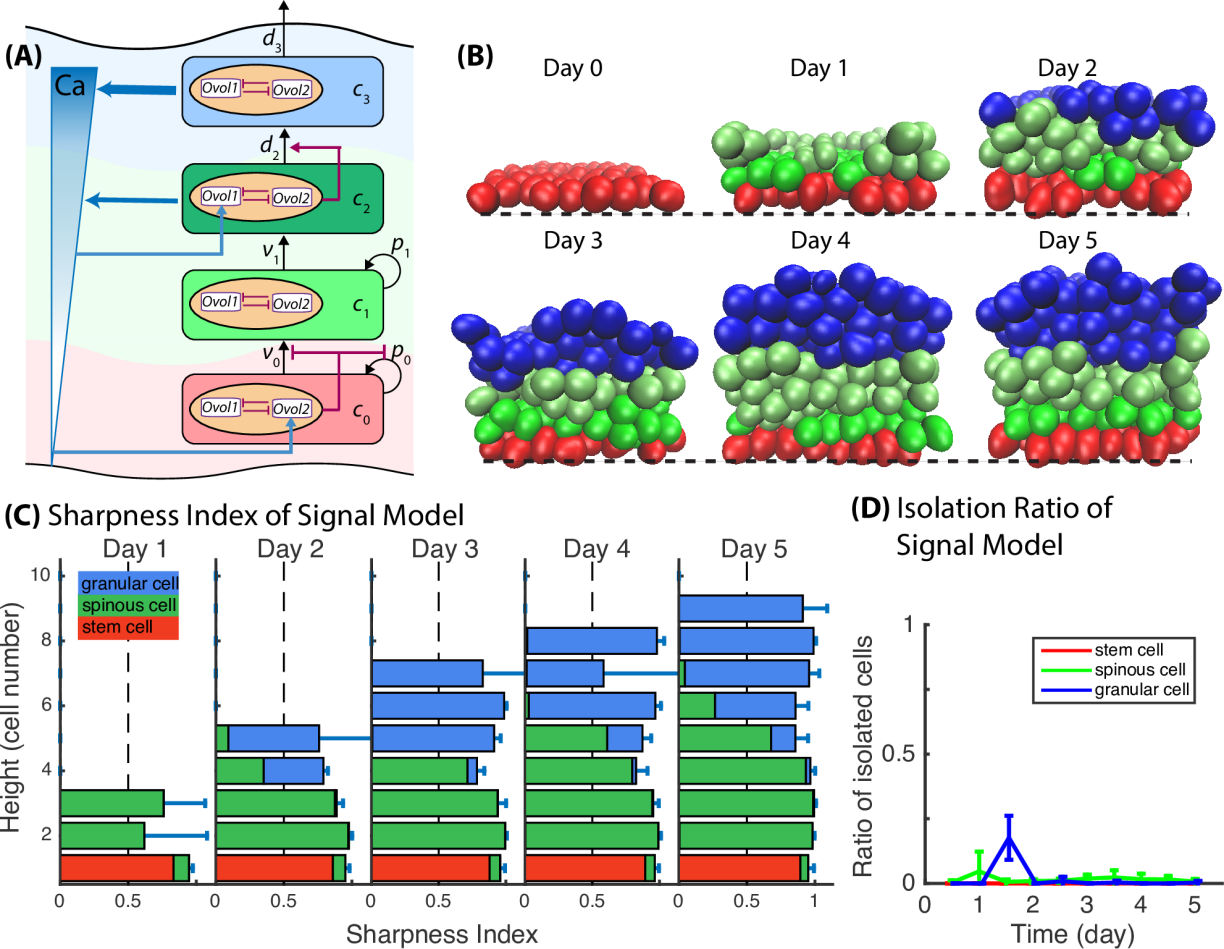
Long-range signaling or morphogen regulation enhances the stratification between spinous and granular cells. (**A**) A schematic diagram shows the calcium regulation on Ovol. (**B**) Time course images of layer formation. Black dashed lines represent the basement membrane. (**C**) Sharpness Index for Signal Model. Bar length represents the value of sharpness index at height in the unit of cell numbers. Red, green and blue stack bars represent the portion of basal stem, spinous and granular cells at height in the unit of cell numbers. (**D**) Isolation Ratio. Mean and standard deviation in 3D model is over an ensemble of 20 simulations. All the parameters used in the simulations are in Table A in S2 Text.

As previous models, which contain only short-range spatial mechanisms such as symmetric/asymmetric division and selective cell adhesion, are not always capable to generate and maintain robust layer formation (Figs 7 and 8, Fig B in S2 Text), we turned to incorporate a global regulation mechanism through the use of a morphogen gradient. Extracellular signals are believed to play a major role in a cell's decision to either proliferate or differentiate. Previous experiments [27] have suggested calcium as a possible extracellular morphogen to regulate epidermal differentiation. In the developing epidermis, *Ovol1* is expressed in suprabasal layers, spinous layers in particular [23], whereas *Ovol2* is expressed predominantly in the basal layer [20]. We conducted simulations exploring the relationship between the epidermal calcium gradient and epidermal development, assuming that (1) both mature spinous cells and terminally differentiated granular cells are able to secret calcium, and the calcium production rate by granular cells are two times higher than that by spinous cells; (2) calcium in turn stimulates *Ovol1* expression inside spinous cells (hence upregulate *d*_2_) and is permissive to *Ovol2* expression inside basal stem cells (hence inhibit *v*_0_ or *p*_0_) (Fig 9A). This is denoted the Signal Model. Simulation results demonstrate that with calcium upregulating *d*_2_, the stratification increases between spinous and granular layers. When spinous cells are misplaced above granular layer (Fig B in S2 Text, scenario 4), or when spinous cells form cluster and intermingle with granular layer (Fig B in S2 Text, scenarios 2), they will sense high calcium concentration secreted by themselves and neighboring granular cells; then with morphogen upregulating *d*_2_, spinous cells speed up differentiation into granular cells, leading to decreased Isolation Ratio of spinous cell and improved layer stratification. Simulation results also demonstrate that in normal stratified tissue, morphogen regulation does not decrease stratification. When spinous cells are adjacent to the lower edge of granular layer, i.e., when they are at the upper edge within the mature spinous layer, they also sense high calcium concentration. However, calcium concentration in well-stratified tissue has near-uniform distribution. Increased differentiation, as a result of calcium upregulating *d*_2_, usually happens among neighboring spinous cells that are adjacent to the granular layer, and the granular cells from differentiation become part of the lower edge of granular layer. This process shifts the boundary between mature spinous cells and granular cells by one cell, and still maintains the boundary under the short-range mechanism of selective cell adhesion. Time course for the Signal Model (Fig 9B), as well as increased Sharpness Index for spinous and granular layers and decreased Isolation Ratio (Fig 9C and 9D), suggest the morphogen regulation mechanism improves the boundary stratification between spinous and granular cells. Analysis based on Ripley’s *K* function also indicates that the Signal Model yields a well-stratified tissue (Fig G in S2 Text). These results indicate that inclusion of long-range spatial mechanism-morphogen regulation enhances the overall epidermis stratification.

**Fig 10:**
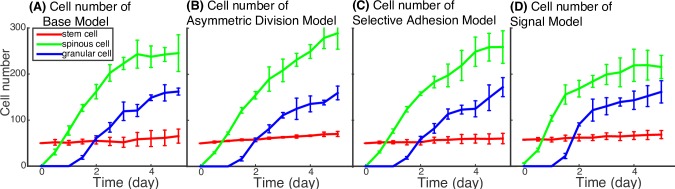
Comparison of cell number for the models. In the Signal Model, negative regulation of basal cell proliferation and positive regulation of spinous cell differentiation into granular cells by the morphogen signaling is able to slow down the cell number’s growth rate and achieve steady state. All the parameters used in the simulations are in Table A in S2 Text.

Signaling regulation also improves epidermis layer size control. In four-stage non-spatial lineage models, the sensitivity analysis and simulation prediction reveal that in order to recapitulate the experimentally observed changes in the basal layer and to prevent the loss of the basal stem cell population, *p*_0_ needs to be greater than ½ (see S1 Text), yielding a trend of ever-growing cell numbers. The 3D spatial model simulation results are consistent with the non-spatial lineage model: in Fig 10A–10C, the pattern that cell number of every cell type keep increasing with time is observed in the first three models (Base Model, Asymmetric Division Model, and Selective Adhesion Model). In the Signal Model (Fig 10D), *p*_0_ is inhibited through Ovol regulation due to increased extracellular morphogen calcium secreted by the growing spinous and granular cell population, and this system is able to approach a steady state where the populations of different cell types are well maintained. The simulation results reveal that precise regulation of proliferation and differentiation through Ovol is required to ensure the proper numbers of differentiated cells at the appropriate time, maintaining overall tissue architecture and homeostasis.

To better understand the interactive components of the multiscale model, additional simulations were performed to investigate functions of each submodel (Section E in S2 Text). The results (Figs I,J of Section E in S2 Text) show how each submodel works and how interactions among the submodels together determine the final outcome (i.e. the overall behavior of the multiscale method). First, based on the Signal Model, the parameter values of *p*_0_, *v*_0_, *p*_1_, *v*_1_, *d*_2_ or *d*_3_ are varied to study tissues with different cell numbers and cell proportions. If we decrease *p*_0_, *v*_0_ or *p*_1_, or increase *v*_1_, *d*_2_ or *d*_3_, the cell number will decrease, and the spatial mechanisms still forms stratified tissue. If the total cell number increase is within a certain range, the asymmetric division, selective adhesion and signaling mechanism are still able to stratify the whole tissue. If the total cell number increases too fast (large *v*_0_, small *v*_1_, *d*_2_ or *d*_3_) or becomes too large (large *p*_0_, *p*_1_), the spatial mechanisms will not be sufficient to form the proper pattern (Fig I in S2 Text). This observation indicates that the spatial mechanisms are relatively robust for pattern formation and that the regulation of proliferation and differentiation by Ovol is critical to forming a stratified tissue.

Next, the asymmetric division component was reduced to 0 in the Signal Model, with the other parameters kept in an appropriate range. With the regulation of selective adhesion and extracellular morphogen, the cells are still able to form a stratified tissue. However, symmetric division will yield cells distributed less regularly than for asymmetric division. As a result, even with other spatial regulation mechanisms, the tissue always presented with uneven boundaries between different cell layers (Fig J in S2 Text). The outcome is similar as scenarios 1,2 in Fig B in S2 Text, but with a much higher frequency. This suggests that without asymmetric division, other spatial mechanisms fail to produce even and flat layers, consistent with the previous analysis of the functions of selective adhesion and extracellular morphogens. The results demonstrate that asymmetric division is critical to forming even and flat layers within the epidermis tissue.

Overall, we find that the results presented here are relatively robust, and that appropriate coupling of the behavior of each submodel–which we used to explore the biological observations–is critical to the overall result.

## Discussion

The simulations of the non-spatial cell lineage model on Ovol have shown that transcription factor regulation of proliferation and differentiation during lineage progression is crucial for the development and maintenance of the epidermal tissue. Using 3D multiscale model to incorporate the multistage cell lineage model and the spatial regulations, we discovered that basal cell asymmetric division, selective intercellular adhesion-derived aggregation, and the influence of morphogen regulation are responsible for both epidermis layer size control and layer pattern formation. Most importantly, simulation results mirror experimental data [9, 10, 23].

Selective adhesion is important for epithelial tissues and many other simple tissues derived from precursor cells. This mechanism enables the cells to be selectively connected with the extracellular matrix, or/and other cells. Selective adhesion is also important in dynamic tissue developmental involving cell migration. Intercellular adhesion in the mammalian epidermis is thought to be mediated in part by cadherins (e.g., E-cadherin)—adhesive components of the adherens junctions. Our model assumes that the accumulating cells do not simply remain passively stuck together; instead they adjust adhesion progressively based on cell types. Our results demonstrate that tissue architecture is generated and actively maintained by selective cell adhesions.

The multiscale model contains several submodels that are connected to determine the overall behavior of the system. Specifically, 1) the multistage cell lineage submodel was designed to be constrained by experimental data (Tables 1–3), and we found that the population size and proportion of each cell type is controlled by this submodel through a gene regulatory network; 2) We systematically explored the effect of symmetric vs. asymmetric division on the overall multiscale model. The asymmetric cell division was found to be critical to robust basal-suprabasal boundary formation, consistent with our previous work [17, 28] on the effects of symmetric vs. asymmetric divisions in different biological systems; 3) The submodel for the selective adhesion, as well as its behavior for various values of other parameters, was explored, showing both its importance in layer stratification and its limitation as a short-range spatial mechanism; and 4) The submodel on morphogen dynamics has two roles: a) providing feedback on controlling the size and the proportion of different cell layers; and b) providing a long-range spatial gradient for signaling from one cell type to another.

The accuracy of the overall multiscale model depends substantially on the functionality and capability of each submodel and how they are connected. Different spatial or temporal scales associated with each model present significant challenges in computation and may need more detailed analytical investigations on their accuracy and integration. To fully explore the interplay among submodels, one may need to investigate wider ranges of (combinations of) parameter values, going beyond the current approach in which parameters were chosen submodel by submodel, Although optimizing each submodel is computationally effective, it is possible that some emerging multiscale features of the system may not be captured using this approach.

It is important to note that a number of aspects of epidermal biology have not been accounted for in our models. For example, we do not account for the various shape and volume of epidermal cells, the resulting function of these cells (e.g. providing a barrier against pathogen infections), and the possibly deformable nature of the basement membrane to which the basal cells adhere. Moreover, the modeling at this point is primarily limited to understanding embryonic epidermal development and does not account for the influence of damage-induced factors on adult epidermis. Future work is needed to study adult repair and regeneration, damage-related effect, cell shape-related functions, and other interesting issues to identify the similarities and differences in basic regulatory principles that govern epidermal development and homeostasis.

Modeling multicellular organisms requires effective tools in describing cells in space, cell-cell interactions, cell-environment interactions, cell division, gene regulatory networks within cells, and communication signals among cells. The computational approach presented in this work provides a good starting point for modeling complex multicellular systems consisting of gene regulatory networks, multiple cell types, cellular lineage hierarchy, cellular mechanics, and their interplays with environments. While each spatial scale and major component of this model, in principle, can be replaced by a method different from this work, the current multiscale coupling allows easy GPU implementation as well as incorporating more complex modules such as dynamic cell fates or more complex gene regulatory networks. One major challenge will be to include multiple multicellular systems that have different spatial and temporal complexities. For example, within the skin, one critical component not modeled in this work is the hair follicle, which introduces another spatial scale and many more cell types. Furthermore, as crosstalk between epidermis and dermis, which consists of many cell types and other functionally important cellular constitutes, is critical to epidermis regeneration, modeling dermis and its interplay with epidermis will naturally require a more complex modeling framework. Our multiscale model of epidermis in this work has laid the foundation for future pursuit in these directions.

## Materials and methods

### Subcellular element model

The subcellular element method divides an individual cell into a set of discrete elements or subcellular elements. Biomechanical forces are then defined as interactions consisting of intracellular dynamics among elements of the same cell and intercellular dynamics between elements of different cells. We assume that the equation of motion of the position vector $Y_{a_{i}}$ of element *a*_*i*_ for cell *i* is (similar to [21, 28])

$$\frac{dY_{a_{i}}}{dt}=-\nabla_{a_{i}}\sum_{i\in I}V_{c(i),c(j),t(i),t(j)}\left(\left|Y_{a_{i}}-Y_{\beta_{j}}\right|\right)-\nabla_{a_{i}}\sum_{i\in I}V_{external}\left(Y_{a_{i}}\right)$$ (4)

where *I* is the set of all elements in the system, *V* is a pairwise force interaction between elements *a*_*i*_ and *β*_*j*_, *V*_*external*_ is any external force that affects that element, *c*(*i*) and *t*(*i*) represent the cell type and the element type, respectively. The pairwise force *V* encompasses both intra- and inter-cellular forces. In the absence of external forces, the intra-cellular forces will scatter the inner elements to the minimum energy configuration with a roughly spherical shape of preferred size. That size is determined by the rest length *r*_0_ for *V*_*intra*_, defining a volume of sorts for the cell. All elements within a cell interact according to the spring potential

$$V_{intra}=\mu \frac{{\left(r_{ij}-r_{0}\right)}^{2}}{2},$$ (5)

where *r*_*ij*_ is the distance between element *i* and element *j* of the same cell and *r*_0_ is a rest length. The inter-cellular force interactions are described by Lennard-Jones type potentials

$$V_{inter}=F_{a,b}\varepsilon \;\left({\left(\frac{\sigma }{\left|r_{ij}\right|}\right)}^{12}-{\left(\frac{\sigma }{\left|r_{ij}\right|}\right)}^{6}\right),$$ (6)

where *r*_*ij*_ is the distance between element *i* and element *j*. The parameter *ε* determines the strength of interaction. *F*_*a*,*b*_ represents the intercellular adhesion strength: *F*_*a*_ for the same type cells and *F*_*b*_ for the different type cells. *σ* is the equilibrium separation where the inter-element potential is zero and two elements are at relative balance position. If the distance between two elements is smaller than *σ*, they experience a repulsion force to prevent overlap of the cell bodies. When the distance between the elements is greater than *σ*, but less than a cutoff value, an attraction exists between the elements. Beyond this cut-off value, we assign zero interaction between cells. These medium range interactions are designed to represent the surface interactions of cadherin-mediated cell-cell adhesion. The adherent force between cell elements and the basement membrane is defined by

$$V_{external}(Y_{\alpha_{i}})=\frac{\varepsilon_{external}}{\left|r_{i}\right|}.$$ (7)

Here, *ε*_*external*_ is the strength of external force, and *r*_*i*_ is the distance between element *i* and the basement membrane. This force has a cut-off distance of half the rest diameter of a cell, to ensure that only the elements “attached” to the basement membrane experience the attraction.

### Coupling of subcellular element model and morphogen diffusion-reaction model in 3D hybrid model

**Fig 11:**
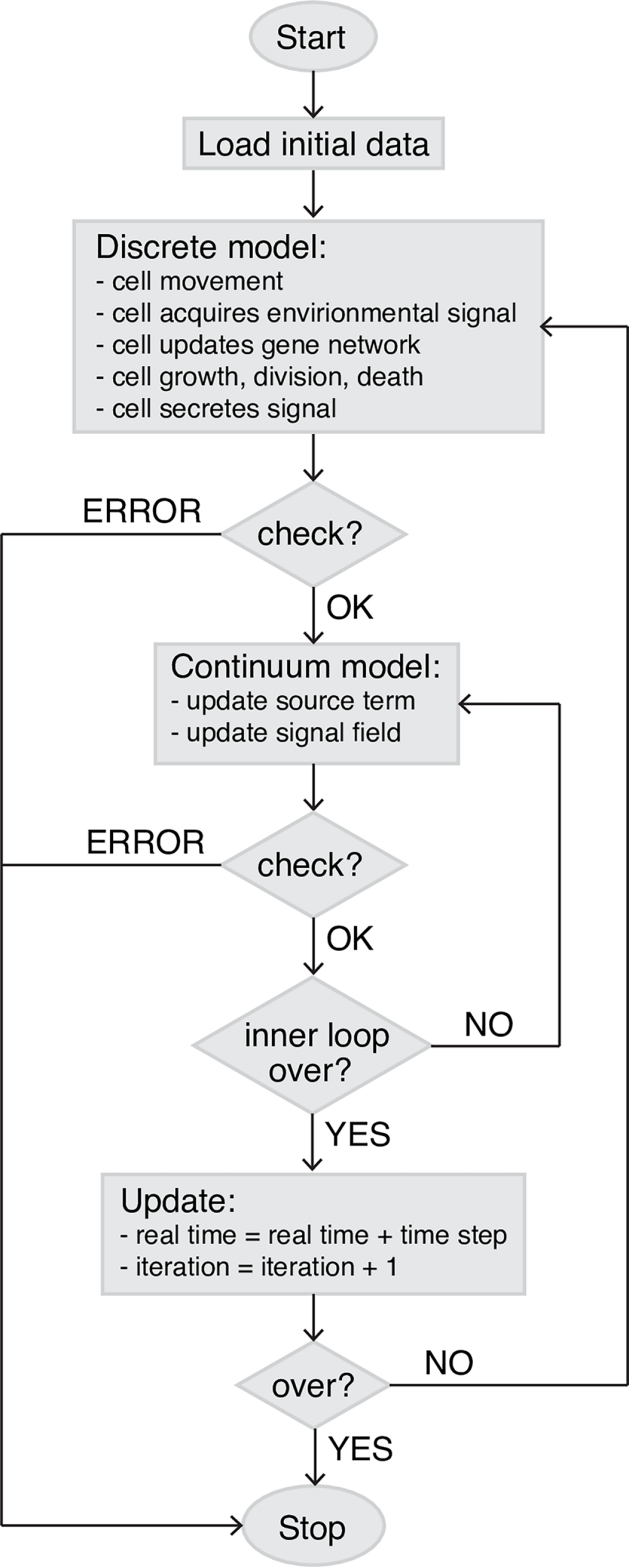
Flowchart of event coordination in the multiscale modeling approach.

To couple cell dynamics and signaling pathway, a regular, rectangular grid for chemical diffusion is superimposed on subcellular element model domain such that each cell element can find its index coordinates of the chemical field. Each simulation time step consists of a substep of subcellular element model followed by a substep of evolution of states of PDEs. During the substep of the cell-based subcellular element model, cells move to a new location, undergo growth and division, make lineage decisions, and produce signals, which modifies the local signal field. An interpolation operator is used to project concentration of signals generated from the subcellular element model domain to the PDE grid blocks: Each element of a cell secrets a constant amount of signal into the PDE grid it locates based on the element coordinates, then the locally produced signal is incorporated into chemical diffusion equation as source term to update the signal field. When cell makes division decision, it will sense the local signal level: each element will record the signal concentration from the PDE grid it is in, and compute a linear combination of the information as the local signal level for cell division decision. During the substep of the PDEs, steady state of signal field is obtained for lineage classification. When updating the chemical diffusion field, diffusivity is based on local element number density. For the diffusion coefficient of the target grid, we first get the element number density of the grid itself and its first degree neighbor grids in 3D, then the diffusivity in each grid is the reciprocal of count density times baseline diffusivity, then the diffusion coefficient of the target grid is just average over the diffusivity of itself and all first degree neighbor grid blocks. In such a way, we defined a simple rule to model the particle density influence on diffusivity. To solve Eq 3, we apply a second-order central difference for the spatial derivatives, and a forward Euler scheme to the temporal discretization. Step size in space is chosen to be 1 *μm*. For each step of chemical diffusion evolution, the chemical field is updated for 1000 times, giving dt = 0.0036s for chemical equation updates. A flowchart (Fig 11) is listed here for event coordination.

### GPU implementation

The code is written in OpenCL allowing it to take full advantage of current advances in parallel processing. The most computationally intensive component of this method is computation of cell dynamics, which is an N-body simulation involving all-pairs approach to compute the all pair-wise force interactions among cell elements. It scales as *O*(*n*^2^) where *n* is the total number of sub-cellular elements in the system. This step is a compute-intensive part but also highly parallel and suitable for GPU application. We followed [17, 21, 29] to provide a parallel implementation of SEM, and include memory layout of data structures and functional decomposition for efficient implementation. Here, the highly-parallel parts like cell dynamics, cell lineage decision and PDE evolution are handled on GPU, while the less frequent activities like cell growth and division are handled outside of the GPU in the CPU code, which helps to minimize the communication between CPU memory and GPU memory as much as possible.

### Sharpness Index for layer boundaries

To quantify the level of stratification of epidermal layers, we introduce the definition of Sharpness Index as a function of layer height. The simulation results are firstly divided into several slices of about one cell size thickness along z-axis. Then each slice is processed into a 10 * 10 pixel image, where each pixel is 0, 1, or 2 to represent that this pixel is occupied by basal stem cells, spinous cells or granular cells, respectively. Image sharpness measure is calculated as one minus the mean square of the horizontal and vertical derivatives, evaluated as finite differences [30]:

$$SI\left(z,g,I\right)=1-\frac{1}{2\#(I)}\sum_{(x,y)\in I}{\left(g\left(x+1,y\right)-g\left(x-1,y\right)\right)}^{2}+{\left(g\left(x,y+1\right)-g\left(x,y-1\right)\right)}^{2},$$ (8)

where *I* is the whole image domain except for the image boundaries, *g*(*x*, *y*) represents the pixel value at grid position (*x*, *y*) for slice at height *z*. This measure of image sharpness is not used for absolute sense, but only to measure the relative sharpness of similar images. According to this definition, sharpness measure for layer boundary will always be less than 1, and *SI* = 1 corresponds to an extreme polarization of the tissue.

### Isolation Ratio for same type cell self-aggregation efficiency

To quantify the efficiency of same type cell self-aggregation by selective adhesion and external signaling regulation, we introduce the definition of Isolation Ratio. At some time point, the coordinates of same type cells are extracted from simulation data, and cells are grouped into same clusters if they are in touch with each other. Then one cluster is marked as the target cluster if it contains the largest number cells and locates at the appropriate position. And the Isolation Ratio is the proportion of the number of cells out of the target cluster over the total cell number. According to this definition, Isolation Ratio = 0 corresponds to a perfect self-aggregation situation.

### Immunofluorescence

Back skins were freshly frozen in optimal cutting temperature (OCT) compound (Tissue Tek), sectioned (8 μm), fixed with 4% paraformaldehyde, and stained using a rabbit anti-K14 antibody at 1:1000 dilution (gift from Julie Segre, National Institute of Health, Bethesda, MD).

### Microarray analysis

Total RNA was extracted from skin using TRIzol reagent (Invitrogen) according to the manufacturer's instructions. One μg of total RNA was reverse transcribed into cDNA using the High Capacity cDNA Reverse Transcription Kit (Applied Biosystems) according to the manufacturer's instructions. Hybridization of arrays (GeneChip Mouse Exon 1.0 ST Array; Affymetrix) was performed in duplicate using independent biological samples. Affymetrix GeneChip Analysis Suite software (MAS5.0) was used to generate raw data, and genes with normalized expression levels over detection threshold were called and analyzed.

### Mice

*K5-tTA;TRE-Ovol2* (*Ovol2* overexpression, *Ovol2* BT), *Ovol1^-/-^;Ovol2^f/-^;K14-Cre* (*Ovol1/Ovol2* double knockout, *Ovol* DKO), *Ovol2^f/-^;K14Cre* (*Ovol2* knockout, *Ovol2* SSKO), and *Ovol1^-/-^* mice have been described previously by [10]. All experiments have been approved, and conform to the regulatory guidelines of the University of California-Irvine International Animal Care and Use Committee.

## Supporting information

S1 Text Multi-stage non-spatial cell lineage model. (PDF)

S2 Text Spatial multiscale model. (PDF)
