# Supplementary material for: Multiscale modeling of layer formation in epidermis
Source: PLoS Comput Biol. 2018 Feb 26;14(2):e1006006. doi: 10.1371/journal.pcbi.1006006 (PMC5843350; doi:10.1371/journal.pcbi.1006006)
Supplement: S1 Text — (PDF) [file pcbi.1006006.s001.pdf]

Multiscale modeling of layer formation in epidermis

Huijing Du[1], Yangyang Wang[2,3], Daniel Haensel[3,4], Briana Lee[3,4], Xing Dai[3,4], Qing Nie[2,3,*]

[1]Department of Mathematics, University of Nebraska-Lincoln, Lincoln, NE 68588, USA

[2]Department of Mathematics, University of California Irvine, Irvine, CA 92697, USA

[3]Center for Complex Biological Systems, University of California Irvine, Irvine, CA 92697, USA

[4]Department of Biological Chemistry, School of Medicine, University of California Irvine, Irvine, CA 92697, USA

[*]Co-corresponding: qnie@math.uci.edu

S1 Text: Multi-stage non-spatial cell lineage model

A. A four-stage epidermal lineage model

Simplifying epidermal development into a cell lineage model of four different cell stages, the equations governing the size of the cell populations in the epidermis are

$$\frac{dC_0}{dt} = (2p_0 - 1)v_0 C_0,$$
$$\frac{dC_1}{dt} = 2(1 - p_0)v_0 C_0 + (2p_1 - 1)v_1 C_1,$$
$$\frac{dC_2}{dt} = 2(1 - p_1)v_1 C_1 - d_2 C_2,$$
$$\frac{dC_3}{dt} = d_2 C_2 - d_3 C_3,$$

where C_0, C_1, C_2, and C_3 represent quantities of K14$^+$ basal cells, K1$^+$ proliferative spinous cells, K1$^+$ terminally proliferated spinous cells, and loricrin$^+$ granular cells, respectively [1]. The definitions of p_0, p_1, d_2, d_3, v_0 and v_1 are as described in the main text. We did not consider cell death in this model, as we did not detect appreciable apoptotic activities in either WT or *Ovol* mutant epidermis.

For the system to achieve a steady state, the requirement $p_0 \to 1/2$ as $t \to \infty$ must be maintained to prevent exponential growth or the extinguishment of the stem cell population and of the tissue as a whole. When $p_0 > 1/2$, dC_0/dt is positive, growth of the basal layer occurs. On the other hand, when $p_0 < 1/2$, dC_0/dt is negative, the basal layer shrinks in size.

Our study is primarily focused on development of the epidermis prior to full maturation. In this scenario, the epidermal lineage system may not reach a steady state, and growth of the basal layer occurs. As a result, we studied the system at a dynamic state after approximately 50 cell cycles (corresponding to $t = 35$) and took values for p_0 exceeding $\frac{1}{2}$.

B. Sensitivity analysis

(1) A sensitivity analysis reveals that as either p_0 or v_0 increases, the sizes of the basal, spinous and granular layers increase (Fig A). No other parameters affect the size of the basal layer since p_0 and v_0 are the only parameters describing behavior of cells in the basal layer. As p_0 or v_0 increases, both the spinous and granular layers increase in size. But the gradient of the basal layer growth behaves differently as p_0 or v_0 increases.

(2) An increase in p_1 prompts an increase in both the spinous layer and the granular layer. However, an increase in v_1 prompts an increase in the granular layer while having little effect on the size of the spinous layer.

(3) Lastly, an increase in d_2 leads to an increase in the size of the spinous layer while the size of the granular layer decreases. And an increase in d_3 prompts a decrease in the granular layer while having little effect on the size of the spinous layer.

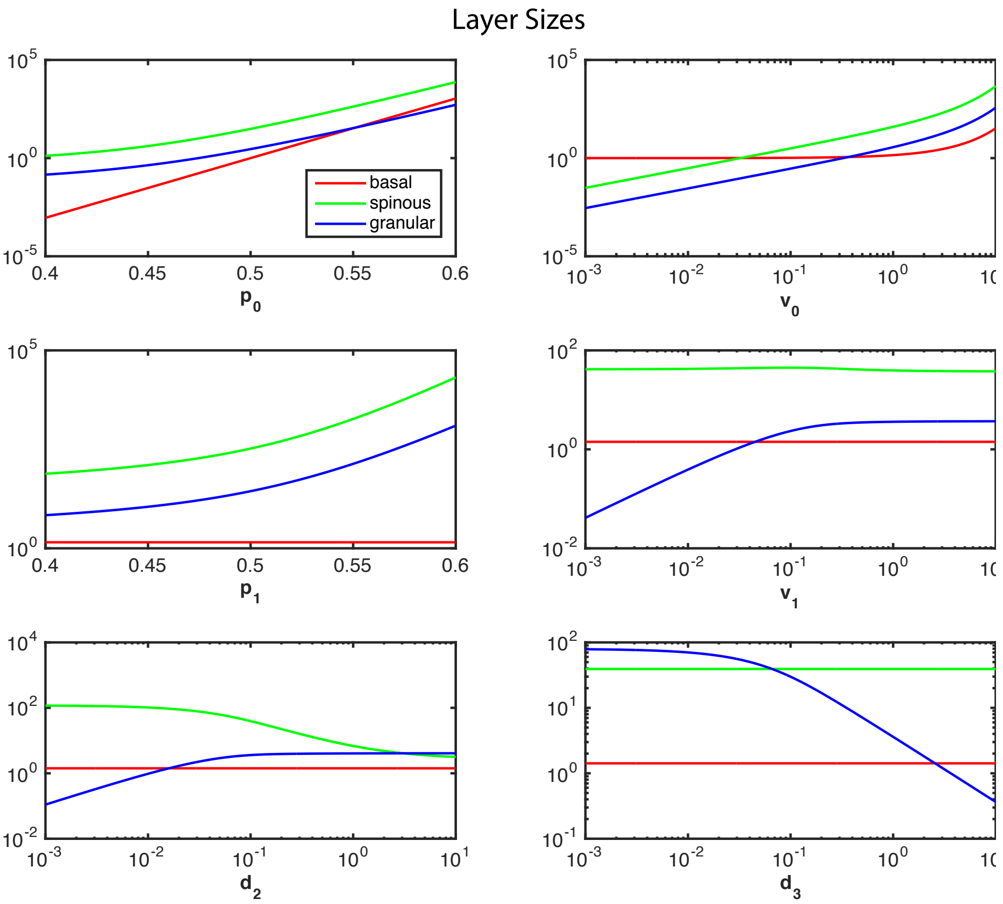

Figure A: Dependence of cell population sizes of different epidermal layers on p_0, v_0, p_1, v_1, d_2 and d_3 values (at time t=35). The sizes of the basal, spinous, and granular layers are given by C_0, C_1+C_2, and C_3, respectively. Corresponding parameters chosen are: p_0=0.515, p_1=0.215, v_0= v_1=1, d_2=0.1, and d_3=1. Initial conditions: C_0(t=0)=1 and C_1(t=0)= C_2(t=0)= C_3(t=0)=0.

C. Incorporating *Ovol* into the lineage model

Based on the skin phenotypes of gain- and loss-of function *Ovol* mutants (*Ovol1*$^{-/-}$, *Ovol2* SSKO, *Ovol2* BT, and *Ovol* DKO), we hypothesize that Ovol1 and Ovol2 hold the potential to down-regulate p_0 (i.e., promote K14$^+$ to K1$^+$ transition), p_1 (i.e., promote growth arrest of K1$^+$ cells), v_0 (i.e., inhibit the proliferation rate of K14$^+$ cells), v_1 (i.e., inhibit the proliferation rate of K1$^+$ cells), and/or up-regulate d_2 (i.e., promote the terminal differentiation of K14$^+$ and K1$^+$ stem/progenitor cells into granular cells).

D. *Ovol*'s inhibition of v_0 and p_0

Since dC_0/dt is linearly dependent upon C_0, the size of the basal layer can be solved by

$$C_0(t) = e^{(2p_0-1)v_0 t}$$

When $p_0 > \frac{1}{2}$, as corresponding to a developmental parameter regime, the basal layer size then increases exponentially with respect to both ν_0 and p_0.

The expanded basal compartment in *Ovol* DKO epidermis suggests increased ν_0 or p_0. When *Ovol1* and *Ovol2* act redundantly to inhibit ν_0 or p_0, the values of ν_0 and p_0 may be near the respective wild-type values in *Ovol1*$^{-/-}$, *Ovol2* SSKO, and *Ovol2* BT epidermis by compensatory mutual repression of *Ovol1* and *Ovol2* while ν_0 and p_0 may be very high in the *Ovol* DKO epidermis due to loss of repression altogether. This would then result in expansion of the basal layer in *Ovol* DKO epidermis while a normal-sized basal layer is maintained in the epidermis of all other mutants.

Following the above analysis, p_0 and v_0 are inhibited upon quantities of *Ovol1* (α) and *Ovol2* (β) expression levels,

$$p_0 = p_{\min} + \frac{p_T}{1 + \lambda\alpha + \mu\beta},$$

$$v_0 = v_{\min} + \frac{v_T}{1 + \omega\alpha + \chi\beta}.$$

E. *Ovol*'s stimulation of d_2

In the sensitivity analysis of the epidermal lineage model, it was noted that only changes in the parameter d_2 result in opposite effects of spinous and granular layer sizes. More specifically, when d_2 is near the range 0.1-1 using the parameters for simulations, a slight decrease in d_2 leads to an expansion of the spinous layers and little change in the size of the granular layers as observed in *Ovol1*$^{-/-}$ epidermis. An increase in d_2 in this range also leads to a shrinking in the size of the spinous layers and less significant changes in the size of the granular layers, as observed in the *Ovol2* BT epidermis. A large decrease in d_2 in this range will then prompt both an increase in the size of the spinous layers and a decrease in the granular layers, as observed in *Ovol* DKO epidermis. Ultimately, these observations prompt the following speculations of how *Ovol* might regulate d_2:

- d_2 is lower in *Ovol1*$^{-/-}$ epidermis than in the wild type.
- d_2 is relatively unchanged in *Ovol2* SSKO epidermis in comparison to the wild type.
- d_2 is higher in *Ovol2* BT epidermis than in the wild type.
- d_2 is significantly lower in *Ovol* DKO epidermis than in the wild type.

Suppose that d_2 assumes the following linear functional form dependent upon quantities of *Ovol1* (α) and *Ovol2* (β) expression levels,

$$d_2 = d_{DKO} + \zeta\alpha + \xi\beta$$

Using this form for d_2, when the *Ovol* expression levels increase, the value of d_2 also increases. Now, if we interpret the speculated levels of *Ovol1* and *Ovol2* in the *Ovol*-deficient and BT epidermis using this functional form and the variables is defined in Table 1, then we have the following relations,

$$\zeta a+\xi b>\xi c,$$
$$\zeta a+\xi b\approx\zeta d,$$
$$\zeta a+\xi b>\zeta g+\xi h,$$
$$\zeta a+\xi b>0,$$

along with the constraint $b>c$ to yield results that may mimic the experimental data. From these formulated relations above, we can deduce that $\xi c>\zeta d$, which indicates that the response that d_2 receives from *Ovol2* in *Ovol1*$^{-/-}$ epidermis is greater than the response from *Ovol1* in *Ovol2*$^{-/-}$ epidermis. This notion suggests that either *Ovol1* represses *Ovol2* in a stronger fashion than *Ovol2* represses *Ovol1* or d_2 is more sensitive to *Ovol2* than to *Ovol1*.

F. Assumption of *Ovol1* represses *Ovol2* in a stronger fashion than *Ovol2* represses *Ovol1* in their mutual inhibition

The expression level of *Ovol1* (α) and *Ovol2* (β) can be represented as

		WT	*Ovol1*$^{-/-}$	*Ovol2* SSKO	*Ovol2* BT	*Ovol* DKO
Experimental findings	*Ovol1*	a	0	d	g	0
	Ovol2	b	c	0	h	0
	Relations		$c>b$	$d>a$	$g<a$, $h>b$	
Model assumption	*Ovol1* (α)	a = 1	0	d = 1.6	g =0.5	0
	Ovol2 (β)	b = 1	c = 1.25	0	h = 10	0

Table A: Summary of *Ovol*'s expression in experiments and models.

G. Two models explaining epidermal phenotypes through two *Ovol* regulations

Our analysis has so far suggested that *Ovol*'s down-regulation of either v_0 or p_0 has the potential to explain the observed changes (or lack of changes) in basal layer size and that *Ovol*'s up-regulation of d_2 may explain the observed changes in spinous and granular layer sizes in *Ovol* mutant skin. Then, we would like to explore all the possible feedbacks of *Ovol* regulation on proliferation and differentiation in Fig B.

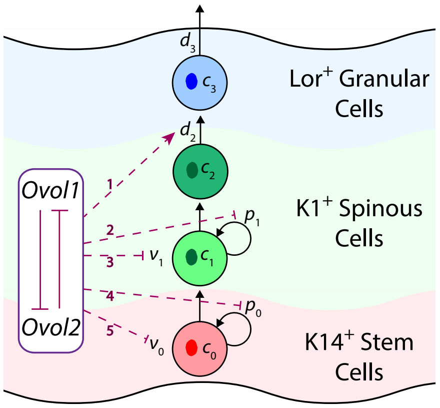

Figure B: A schematic diagram shows *Ovol* regulation of epidermal cell proliferation and differentiation. Red dashed lines represents the potential regulation relationships between the *Ovol* genes and components of the cell lineage model.

$\downarrow p_0 + \uparrow d_2$
$\downarrow p_0 + \uparrow d_2 + \downarrow v_0$
$\downarrow p_0 + \uparrow d_2 + \downarrow p_1$
$\downarrow p_0 + \uparrow d_2 + \downarrow v_1$
$\downarrow p_0 + \uparrow d_2 + \downarrow v_0 + \downarrow p_1$
$\downarrow p_0 + \uparrow d_2 + \downarrow v_0 + \downarrow v_1$
$\downarrow p_0 + \uparrow d_2 + \downarrow p_1 + \downarrow v_1$
$\downarrow p_0 + \uparrow d_2 + \downarrow v_0 + \downarrow p_1 + \downarrow v_1$
$\downarrow v_0 + \uparrow d_2$
$\downarrow v_0 + \uparrow d_2 + \downarrow p_1$
$\downarrow v_0 + \uparrow d_2 + \downarrow v_1$
$\downarrow v_0 + \uparrow d_2 + \downarrow p_1 + \downarrow v_1$

Table B: Summary of all possible combinations of *Ovol* regulation of proliferation and differentiation that can explain epidermal phenotypes in experiments.

All the *Ovol* regulations that can capture the epidermal phenotypes are listed in Table B. This intuitive exploration of feedback loops shows that *Ovol* down-regulation of p_0 or v_0 and its up-regulation of d_2 are the key components (Model 1 and Model 2 below). And with the assistance of other feedback loops, there exist several more complex models (for example Model 3) also capable to reproduce the experimental observation.

<u>Model 1</u>: *Ovol1* and *Ovol2* inhibit p_0 and up-regulate d_2 through the functional forms,

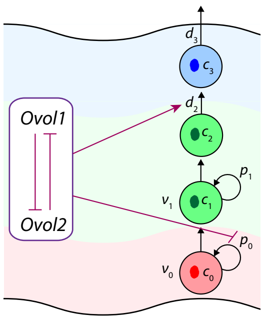

$$p_0 = p_{\min} + \frac{p_T}{1 + \lambda\alpha + \mu\beta},$$

$$d_2 = d_{DKO} + \zeta\alpha + \xi\beta$$

Model 2: *Ovol1* and *Ovol2* inhibit v_0 and up-regulate d_2 through the functional forms,

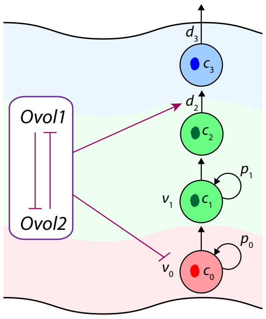

$$v_0 = v_{\min} + \frac{v_T}{1+\omega\alpha+\chi\beta},$$
$$d_2 = d_{DKO} + \zeta\alpha + \xi\beta.$$

Model 3: *Ovol1* and *Ovol2* inhibit v_0 and p_1, and up-regulate d_2 through the functional forms,

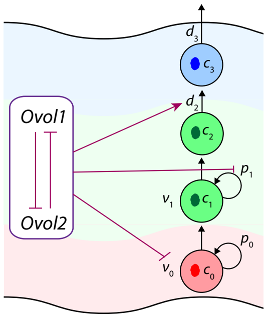

$$v_0 = v_{\min} + \frac{v_T}{1+\omega\alpha+\chi\beta},$$
$$p_1 = p_{\min} + \frac{p_T}{1+\lambda\alpha+\mu\beta},$$
$$d_2 = d_{DKO} + \zeta\alpha + \xi\beta.$$

In all three models we assume that *Ovol1* and *Ovol2* inhibit the expression of one another.

All models can recapitulate the experimentally observed phenotypes of *Ovol*-deficient and -overexpressing epidermis (Tables C, D and E). Note that Model 3 also incorporates inhibition of p_1 from *Ovol1* and *Ovol2*, although the majority of the phenotypes may be explained by inhibition of v_0 and upregulation of d_2 alone as in Model 2.

Model 1	**WT**		***Ovol1*$^{-/-}$**		***Ovol2* SSKO**		***Ovol2* BT**		***Ovol* DKO**	
K14$^+$ basal layer	1.60	—	1.87	—	1.72	—	1.13	—	4.06	↑
K1$^+$ spinous layer	8.18	—	17.50	↑	8.37	—	3.08	↓	169.14	↑
Lor$^+$ granular layer	4.11	—	4.56	—	4.38	—	3.07	↓	1.55	↓
Ovol1 (α)	a = 1		0		d = 1.6		g =0.5		0	
Ovol2 (β)	b = 1		c = 1.25		0		h = 10		0	

Table C: Results from Model 1 with *Ovol*'s down-regulation of p_0 and up-regulation of d_2. Corresponding parameters chosen are: p_1=0.215, v_0=v_1=1, d_3=1, $p_{\min}$=0.5, p_T=0.02, λ=μ=1, d_{DKO}=10^{-2}, ζ=0.5 and ξ=0.25. Simulation ran up to time t = 35.

Model 2	**WT**		***Ovol1*$^{-/-}$**		***Ovol2* SSKO**		***Ovol2* BT**		***Ovol* DKO**	
K14$^+$ basal layer	2.81	—	2.90	—	2.85	—	2.59	—	3.35	↑
K1$^+$ spinous layer	13.37	—	25.78	↑	13.17	—	4.27	↓	174.80	↑
Lor$^+$ granular layer	6.57	—	6.59	—	6.75	—	5.80	↓	1.61	↓
Ovol1 (α)	a = 1		0		d = 1.6		g =0.5		0	
Ovol2 (β)	b = 1		c = 1.25		0		h = 50		0	

Table D: Results from Model 2 with *Ovol*'s down-regulation of v_0 and up-regulation of d_2. Corresponding parameters chosen are: p_0=0.515, p_1=0.215, v_1=1, d_3=1, $v_{\min}$=0.9, v_T=0.25, $\omega=\chi$=1, $d_{DKO}=10^{-2}$, ζ=0.5 and ξ=0.25. Simulation ran up to time t = 35.

Model 3	**WT**		***Ovol1***$^{-/-}$		***Ovol2* SSKO**		***Ovol2* BT**		***Ovol* DKO**	
K14$^+$ basal layer	2.86	—	2.99	—	2.92	—	2.64	—	4.35	↑
K1$^+$ spinous layer	15.97	—	33.52	↑	16.47	—	6.22	↓	472.21	↑
Lor$^+$ granular layer	7.66	—	8.39	—	8.19	—	6.01	↓	4.24	↓
Ovol1 (α)	a = 1		0		d = 1.6		g =0.5		0	
Ovol2 (β)	b = 1		c = 1.25		0		h = 10		0	

Table E: Results from Model 3 with *Ovol*'s down-regulation of v_0 and p_1 and up-regulation of d_2. Corresponding parameters chosen are: p_0=0.515, v_1=1, d_3=1, $v_{\min}$=0.9, v_T=0.5, $\omega=\chi$=2, $p_{\min}=p_T$=0.2, $\lambda=\mu$=1, $d_{DKO}=10^{-2}$, ζ=0.5 and ξ=0.25. Simulation ran up to time t = 35.

**Model 1**	Initial condition	c_0=1, c_1= c_2=c_3=0
	Parameter values	p_1=0.215, $v_0=v_1$=1, d_3=1, $p_{\min}$=0.5, p_T=0.02, $\lambda=\mu$=1, $d_{DKO}=10^{-2}$, ζ=0.5, ξ=0.25
**Model 2**	Initial condition	c_0=1, c_1= c_2=c_3=0
	Parameter values	p_0=0.515, p_1=0.215, v_1=1, d_3=1, $v_{\min}$=0.9, v_T=0.25, $\omega=\chi$=1, $d_{DKO}=10^{-2}$, ζ=0.5, ξ=0.25
**Model 3**	Initial condition	c_0=1, c_1= c_2=c_3=0
	Parameter values	p_0=0.515, v_1=1, d_3=1, $v_{\min}$=0.9, v_T=0.5, $\omega=\chi$=2, $p_{\min}=p_T$=0.2, $\lambda=\mu$=1, $d_{DKO}=10^{-2}$, ζ=0.5, ξ=0.25

Table F: Initial condition and parameter values of the multistage cell lineage model.

SUPPLEMENTAL REFERENCES

1. Lander AD, Gokoffski KK, Wan FY, Nie Q, Calof AL. Cell lineages and the logic of proliferative control. PLoS Biol. 2009;7(1):e15.
