# Supplementary material for: Multiscale modeling of layer formation in epidermis
Source: PLoS Comput Biol. 2018 Feb 26;14(2):e1006006. doi: 10.1371/journal.pcbi.1006006 (PMC5843350; doi:10.1371/journal.pcbi.1006006)
Supplement: S2 Text — (PDF) [file pcbi.1006006.s002.pdf]

Multiscale modeling of layer formation in epidermis

Huijing Du[1], Yangyang Wang[2,3], Daniel Haensel[3,4], Briana Lee[3,4], Xing Dai[3,4], Qing Nie[2,3,*]

[1]Department of Mathematics, University of Nebraska-Lincoln, Lincoln, NE 68588, USA

[2]Department of Mathematics, University of California Irvine, Irvine, CA 92697, USA

[3]Center for Complex Biological Systems, University of California Irvine, Irvine, CA 92697, USA

[4]Department of Biological Chemistry, School of Medicine, University of California Irvine, Irvine, CA 92697, USA

[*]Co-corresponding: qnie@math.uci.edu

S2 Text: Spatial multiscale model

A. Initial condition and boundary condition.

Initially we begin with 50 basal cells randomly placed on the basement membrane, and each cell is set with a random initial time. The domain is set with periodic boundary condition in the horizontal direction, and no flux boundary condition on the basement membrane. For simplicity, we assume that the signal cannot diffuse beyond the top layer of the epidermal tissue. Rather than defining no flux boundary conditions on complex surfaces, we instead extend the computational domain beyond the domain containing the cells and assign the chemical diffusion coefficient to be $D_c=0$ on the extended domain. In addition to simplifying boundary conditions, this also allows the use of a rectangle shaped domain, which allow efficient computation.

**Parameters**	**Values**
Cell diameter	10 microns [1,2]
Basement membrane area	100 microns × 100 microns
Basal cell division cycle	24 hours [3,4]
Element number (N)	20
Intra-cellular potential	μ=2.5, r_0=1.5, no cut-off distance.
Inter-cellular potential	ε=0.05, σ=4.5, cut-off distance is 10.0. $F_a = F_b = 2$ for Base and Asymmetric Division Model; $F_a = 4, F_b = 1$ for Signal Model.
External force	$\varepsilon_{external}$=0.001, cut-off distance is 5.0.
Probability	Use values from Model 1 and 2 of Table F in S1 Text, except $p_{min} = 0.25, q_0 = 0.25, p_1 = 0.2, q_1 = 0.03$ for Asymmetric Division, Selective Adhesion and Signal Model
Calcium production	$\delta_{s1} = 0.005$ for mature spinous cell; $\delta_{s2} = 0.01$ for granular cell
Baseline Diffusion (D)	$10^{-7} cm^2/s$ [5]
Decay (d)	$10^{-3}/s$ [5]
Time step (dt)	3.6s for SEM; 0.0036s for chemical equation

Table A. A list of parameter values used for the 3D model. In cases where parameters are drawn from literature, references are provided.

B. Number of simulation runs.

The 3D model contains stochastic components from the individual cell activity, and we computed the mean and standard deviation of observed quantities. We determined the number of runs of simulations when the values of mean and standard deviation remain relatively stable as the number of runs of simulations increases. We also considered balancing modeling performance (more runs of simulation provide a more accurate prediction) and computational costs (more runs of simulation is more expensive). Fig A below shows Sharpness Index of each model with 25 simulation runs, and the difference of SI between 20 simulation runs (Figs 5,6,9) and 25 simulation runs (Fig A) are small. We found the summary statistics based on 20 simulations provides a “convergent” estimate of the interested quantities, along with affordable computational cost.

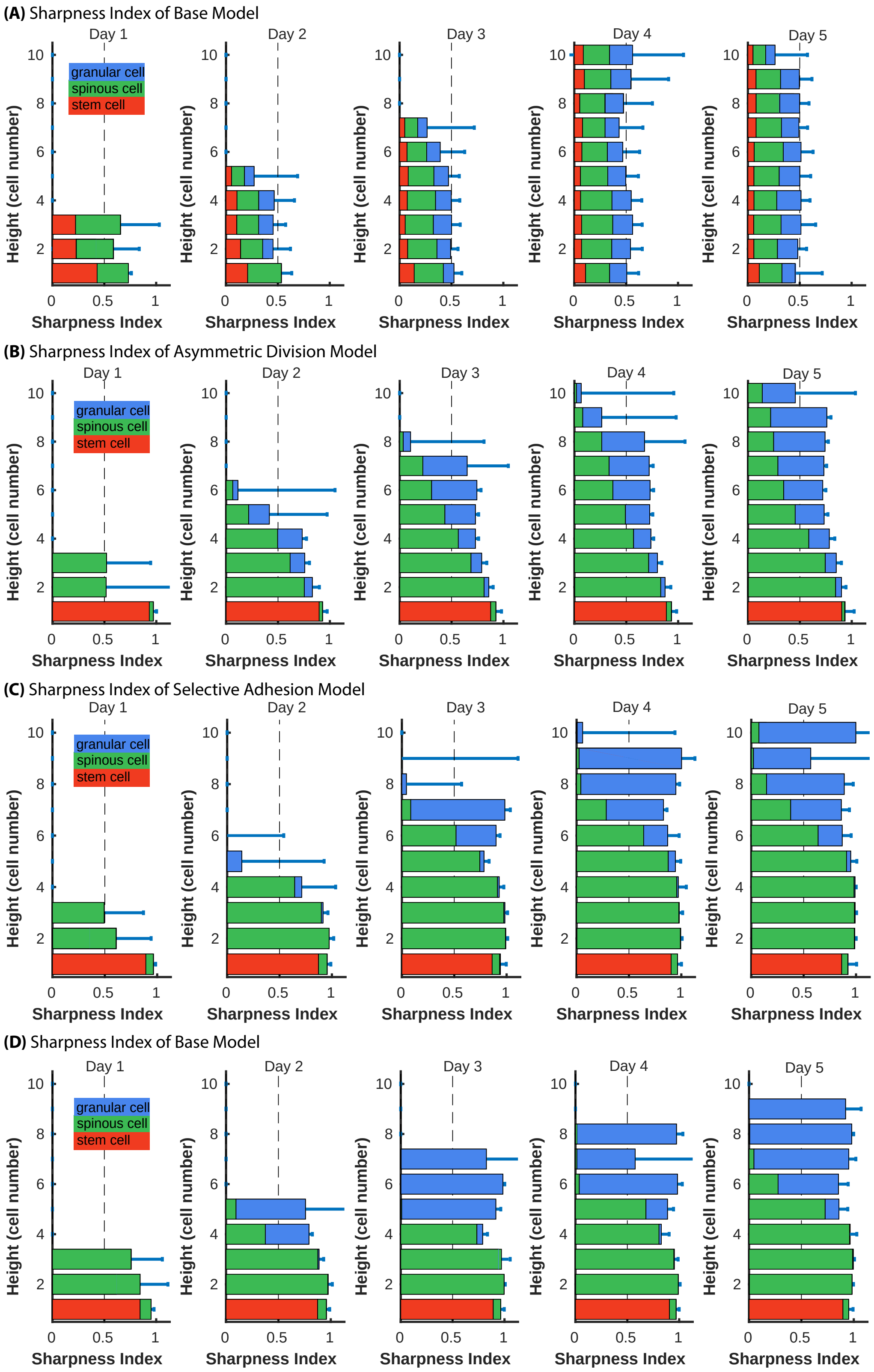

Figure A: SI of each model based on 25 simulation runs. The parameter values are shown in Table A in S2 Text.

C. Investigation of Selective Adhesion Model

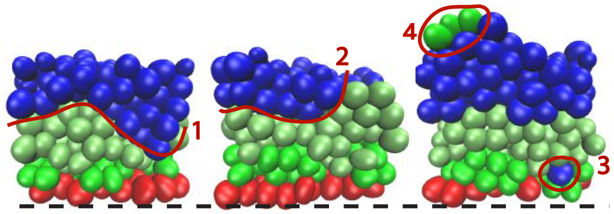

Figure B: Several typical scenarios related with the weakness of selective cell adhesion. Scenario (1): uneven layer boundary; Scenario (2): broken granular layer; Scenario (3): isolated granular cells intermingled with spinous cells; Scenario (4): isolated spinous cells staying on top of the tissue.

D. Stratification measured using Ripley's *K* function

Ripley's *K* function is a good measure to analyze point patterns at multiple distances [6]. Here we use it as an additional measure to investigate the spatial pattern of the same type cells at multiple distances in each slice along the z direction to compare with the investigations using Sharpness Index and Isolation Ratio. We have plotted the Ripley's *K* function for a typical simulation of each model (Fig C-G). The results of Ripley's *K* function show consistency with the analysis using Sharpness Index and Isolation Ratio.

In the Base Model (Fig C), Ripley's *K* function calculations show that every layer along the z-axis is a mixture of three cell types. Basal stem cells form clusters at short cell distance, which is a natural result of self-proliferation. Spinous and granular cells distribute evenly across the tissue. This analysis shows consistency with the results of Sharpness Index and Isolation Ratio (Fig 3, 5AB).

In the Asymmetric Division Model (Fig D), Ripley's *K* function calculations show that basal stem cells distribute regularly in the first layer with attachment to the basement membrane, while spinous and granular cells distribute regularly in the tissue except in the first layer, which is similar to the observation based on Sharpness Index and Isolation Ratio (Fig 4B, 5CD).

In the Selective Adhesion Model, for the good scenario (Fig E) Ripley's *K* function calculations show that the mechanism yields the stratified pattern. However, in the bad scenario (Fig F), there is a cluster of spinous cells above the granular layer. Both cases are consistent with the analysis using Sharpness Index and Isolation Ratio for the Selective Adhesion Model (Fig 6CD, B).

In the Signal Model (Fig G), Ripley's *K* function calculations show that the mechanism yields the stratified pattern, which is consistent with the analysis using Sharpness Index and Isolation Ratio for the Signal Model (Fig 9BCD).

We also checked the pair correlation function for each model, and they provide the similar results. For example, Fig H presents the pair correlation function for a typical simulation of the Signal Model, showing the tissue is stratified and the same type cells are distributed more regularly within their layer. The small peaks when cell pair distances equal 1.5 cell diameter indicates the same type cells tend to cluster at short cell distance, which is likely due to proliferation and selective cell adhesion.

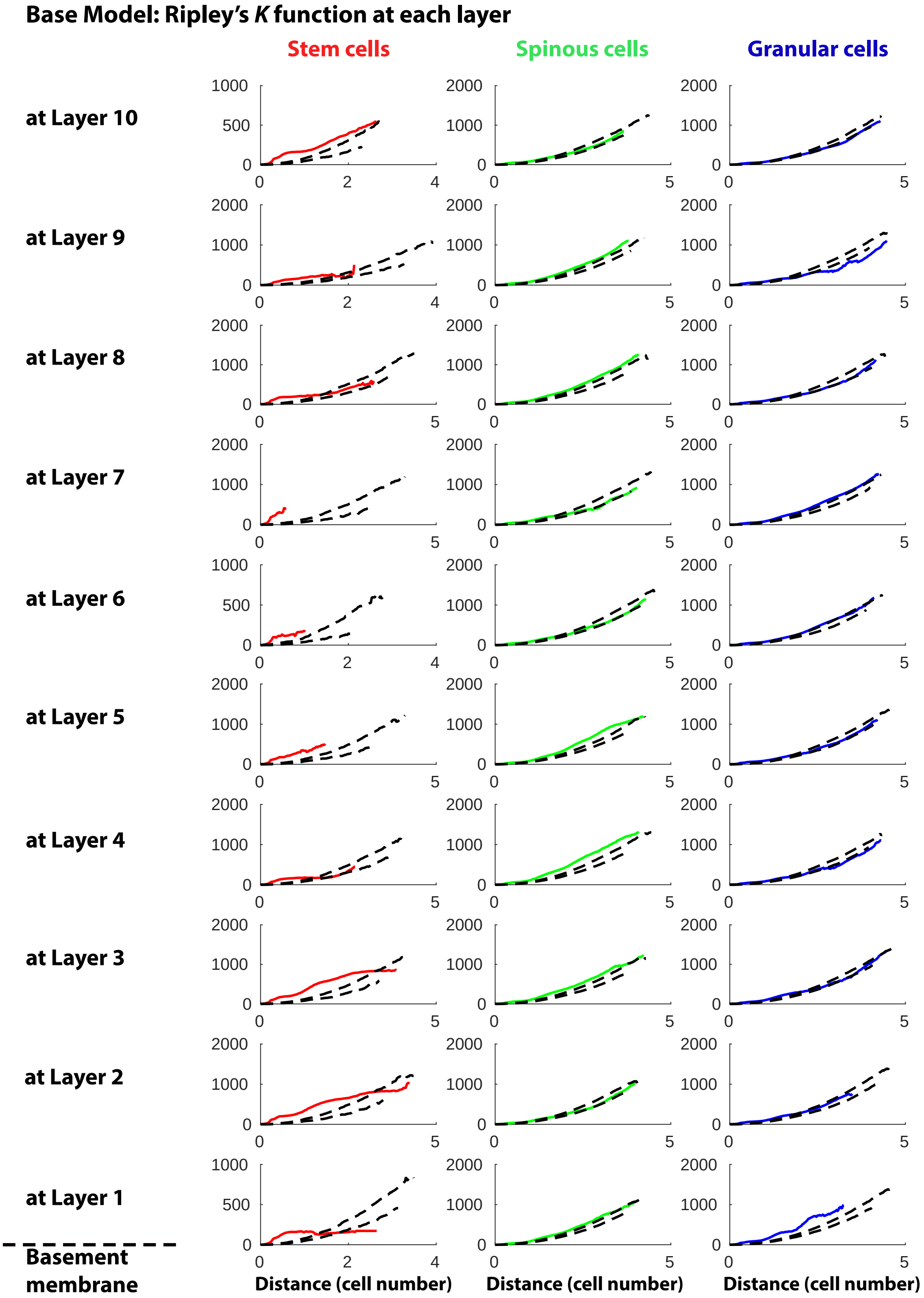

Figure C: Calculations of Ripley's *K* function for the Base Model show that cells of different cell types distribute regularly across the tissue. The parameter values used are shown in Table A in S2 Text.

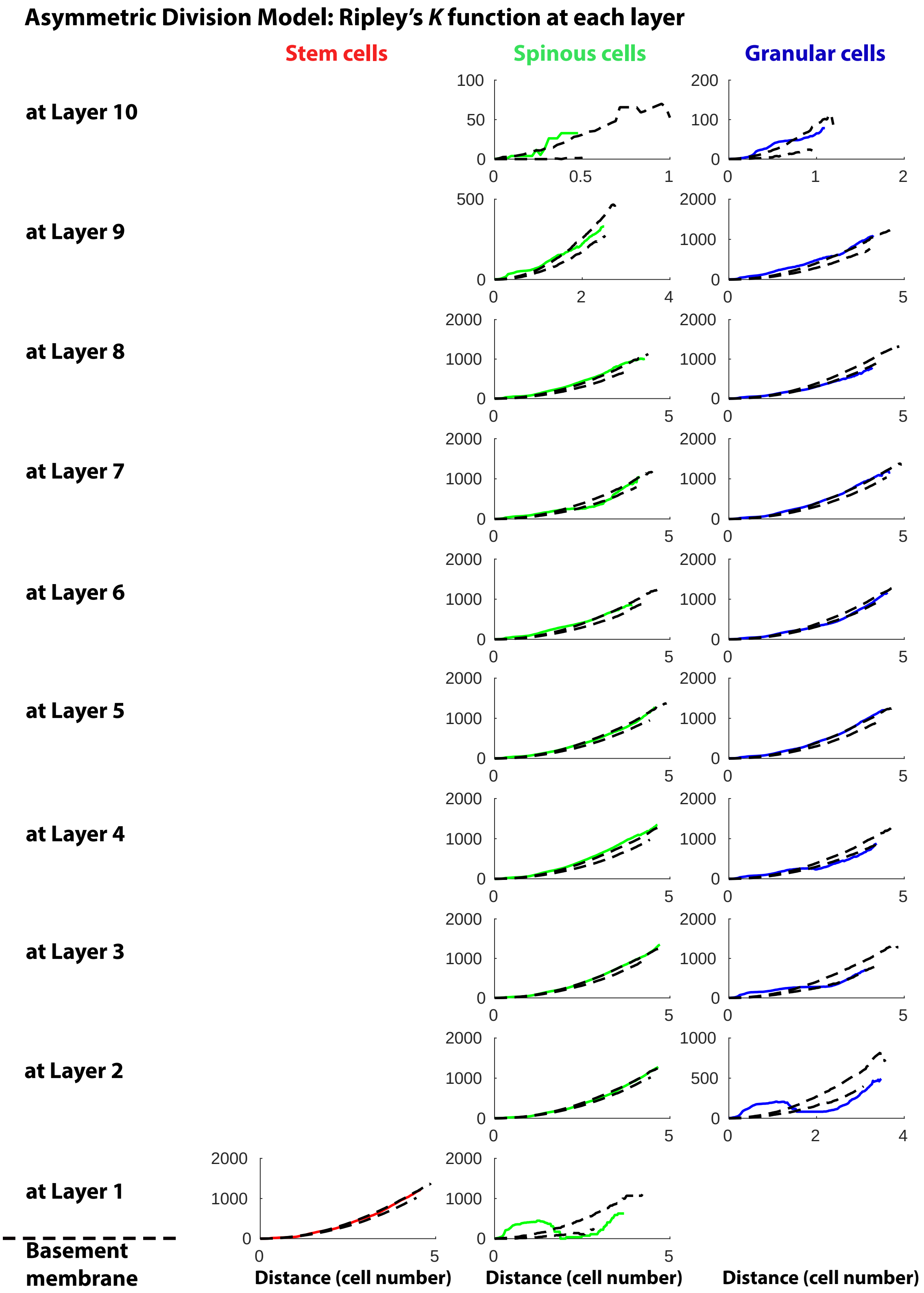

Figure D: Calculations of Ripley's *K* function for the Asymmetric Division Model show that stem cells distribute in the first layer, while spinous and gradular cells distribute regularly in the tissue except the first layer. The parameter values used are shown in Table A in S2 Text.

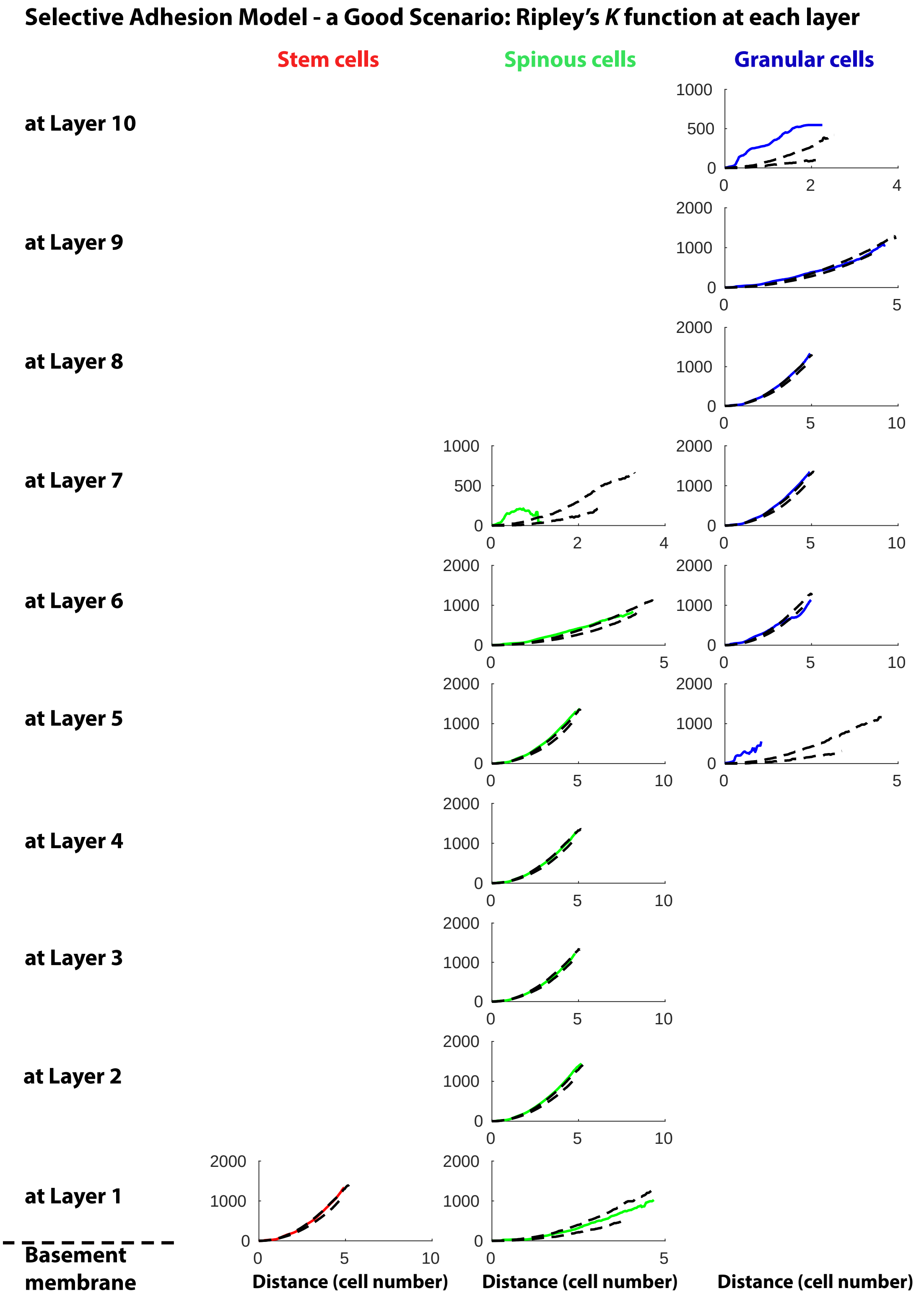

Figure E: Calculations of Ripley's *K* function for the Selective Adhesion Model show that the mechanism works to pattern the tissue. The parameter values used are shown in Table A in S2 Text.

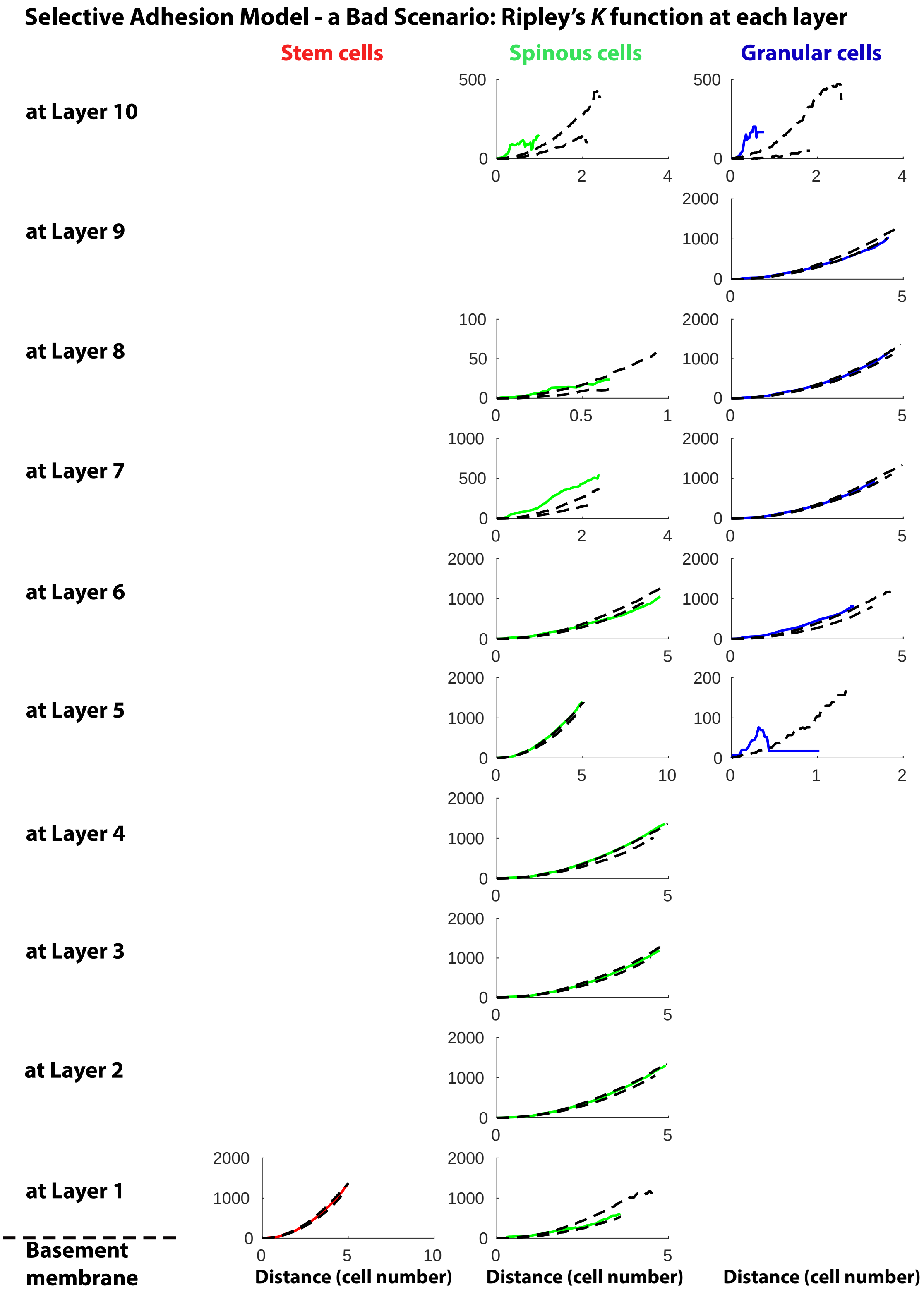

Figure F: Calculations of Ripley's *K* function for the Selective Adhesion Model show the existence of isolated cluster of spinous cells above the granular layer. The parameter values used are shown in Table A in S2 Text.

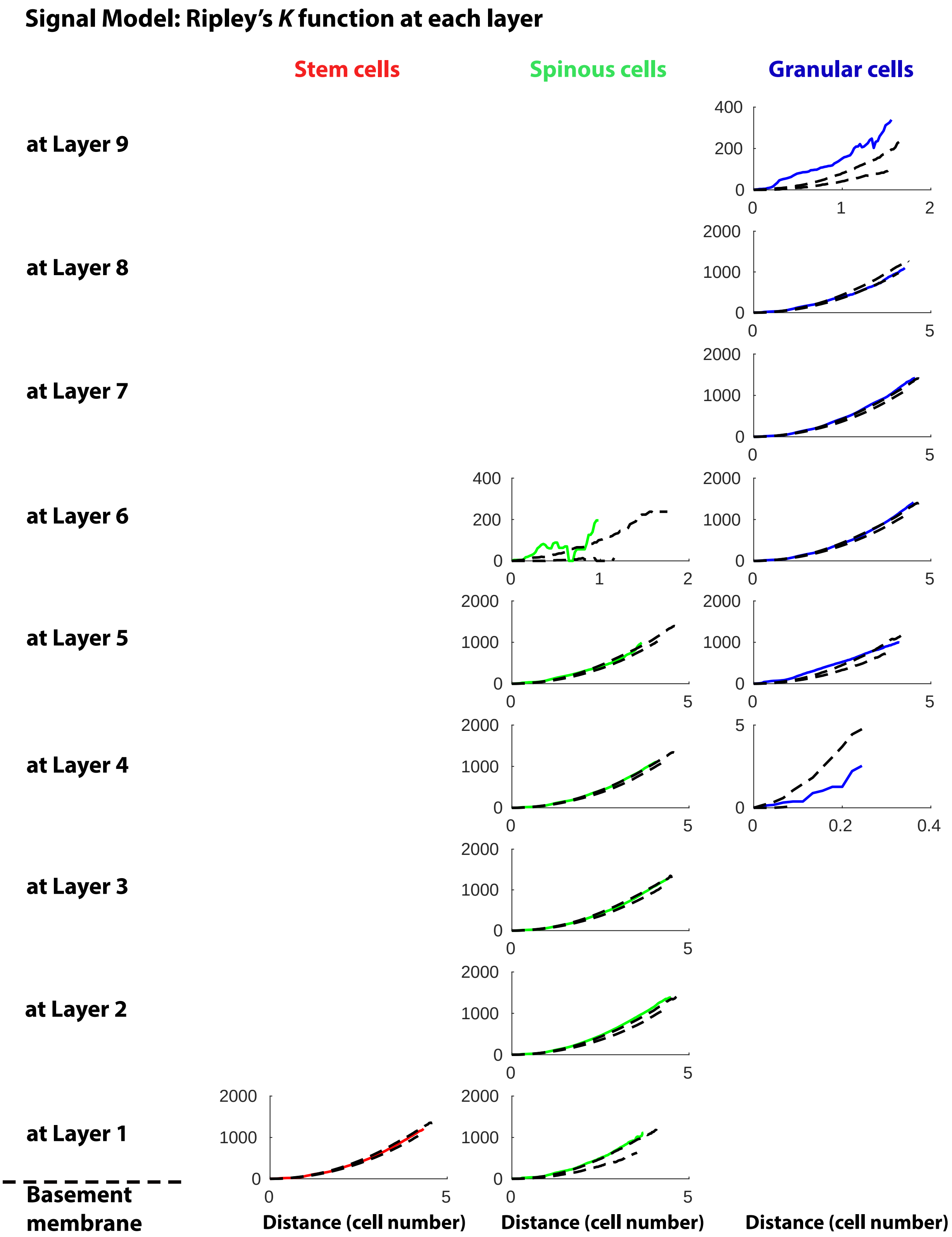

Figure G: Calculations of Ripley's *K* function for the Signal Model show that the mechanism works well to pattern the tissue. The parameter values used are shown in Table A in S2 Text.

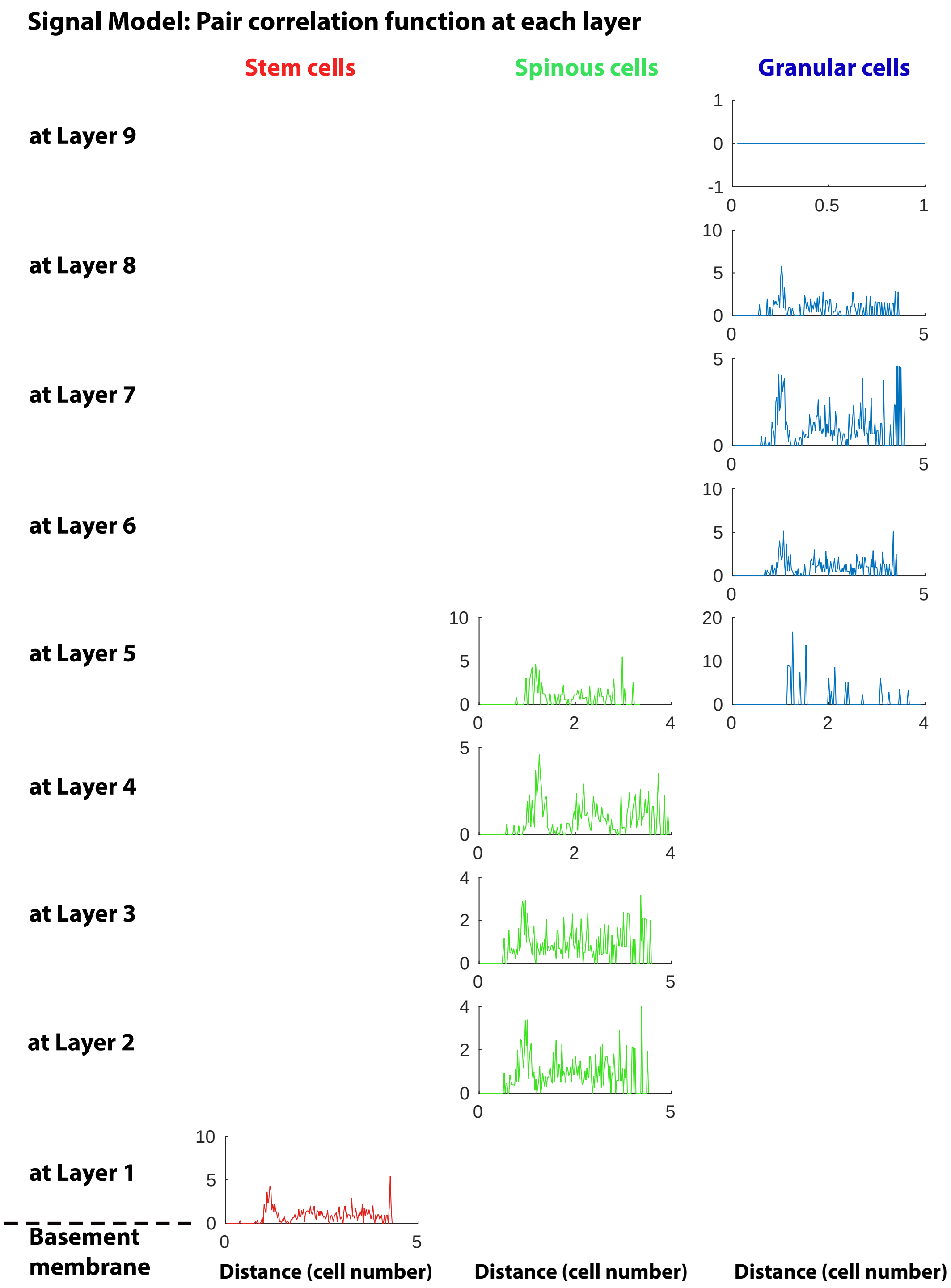

Figure H: A pair correlation function for the Signal Model show that the mechanism works to pattern the tissue. The parameter values used are shown in Table A in S2 Text.

E. Investigation of each submodel of the 3D multiscale model

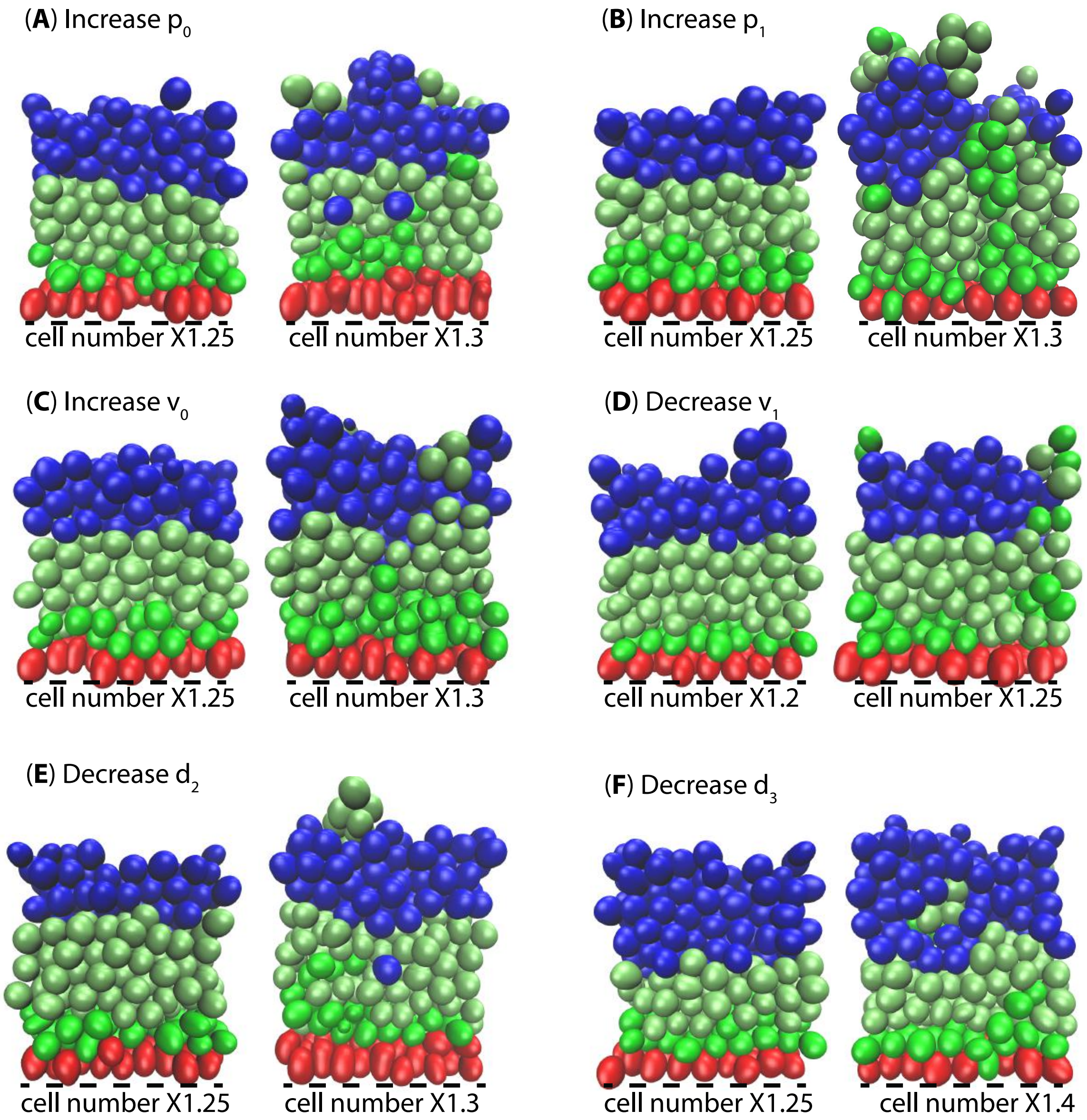

Figure I: Simulations for the Signal Model with varied parameter values. In each panel, the plot on the left is a snapshot at Day 4 for a typical good scenario, while the plot on the right is a snapshot at Day 4 for a typical bad scenario. The parameter values used are shown in Table A in S2 Text.

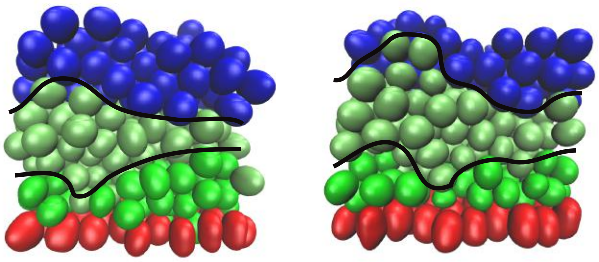

Figure J: A simulation using the Signal Model when the asymmetric division component is reduced to 0. The parameter values used are shown in Table A in S2 Text.

SUPPLEMENTAL REFERENCES

1. Barrandon Y, Green H. Cell size as a determinant of the clone-forming ability of human keratinocytes. Proceedings of the National Academy of Sciences of the United States of America. 1985;82(16):5390-4.
2. Lee B, Villarreal-Ponce A, Fallahi M, Ovadia J, Sun P, Yu Q-C, et al. Transcriptional Mechanisms Link Epithelial Plasticity to Adhesion and Differentiation of Epidermal Progenitor Cells. Developmental Cell. 2014;29(1):47-58.
3. Clausen OPF, Kirkhus B, Thorud E, Schjølberg A, Moen Eva, Cromarty A. Evidence of Mouse Epidermal Subpopulations with Different Cell Cycle Times. Journal of Investigative Dermatology. 1986;86(3):266-70.
4. Clausen OPF, Kirkhus B, Schjølberg AR. Cell Cycle Progression Kinetics of Regenerating Mouse Epidermal Cells: An In Vivo Study Combining DNA Flow Cytometry, Cell Sorting, and [3H]dThd Autoradiography. Journal of Investigative Dermatology. 1986;86(4):402-5.
5. Lander AD, Nie Q, Wan FYM. Do morphogen gradients arise by diffusion? Dev Biol. 2002;2(6):485-96.
6. Stoyan DS, H. Fractals, Random Shapes and Point Fields. Methods of Geometrical Statistics.: Wiley, UK; 1994.
